# Targeting CEBPA to restore cellular identity and tissue homeostasis in pulmonary fibrosis

**DOI:** 10.1172/jci.insight.175290

**Published:** 2024-07-16

**Authors:** Qi Tan, Jack H. Wellmerling, Shengren Song, Sara R. Dresler, Jeffrey A. Meridew, Kyoung M. Choi, Yong Li, Y.S. Prakash, Daniel J. Tschumperlin

**Affiliations:** 1 The Hormel Institute, University of Minnesota, Austin, Minnesota, USA.; 2 Department of Physiology and Biomedical Engineering, Mayo Clinic, Rochester, Minnesota, USA.; 3 Department of Anesthesiology and Perioperative Medicine, Mayo Clinic, Rochester, Minnesota, USA.

**Keywords:** Pulmonology, Stem cells, Adult stem cells, Fibrosis, Pulmonary surfactants

## Abstract

Fibrosis in the lung is thought to be driven by epithelial cell dysfunction and aberrant cell-cell interactions. Unveiling the molecular mechanisms of cellular plasticity and cell-cell interactions is imperative to elucidating lung regenerative capacity and aberrant repair in pulmonary fibrosis. By mining publicly available RNA-Seq data sets, we identified loss of CCAAT enhancer-binding protein alpha (CEBPA) as a candidate contributor to idiopathic pulmonary fibrosis (IPF). We used conditional KO mice, scRNA-Seq, lung organoids, small-molecule inhibition, and potentially novel gene manipulation methods to investigate the role of CEBPA in lung fibrosis and repair. Long-term (6 months or more) of *Cebpa* loss in AT2 cells caused spontaneous fibrosis and increased susceptibility to bleomycin-induced fibrosis. *Cebpa* knockout (KO) in these mice significantly decreased AT2 cell numbers in the lung and reduced expression of surfactant homeostasis genes, while increasing inflammatory cell recruitment as well as upregulating *S100a8/a9* in AT2 cells. In vivo treatment with an S100A8/A9 inhibitor alleviated experimental lung fibrosis. Restoring CEBPA expression in lung organoids ex vivo and during experimental lung fibrosis in vivo rescued CEBPA deficiency–mediated phenotypes. Our study establishes a direct mechanistic link between CEBPA repression, impaired AT2 cell identity, disrupted tissue homeostasis, and lung fibrosis.

## Abstract 2

<strong>CEBPA deficiency causes loss of AT2 cells and induces profibrotic response, which provides new possibilities for lung fibrosis treatment by restoring insufficient CEBPA expression.</strong>

## Introduction

Fibrosis is the end-stage pathological outcome of many chronic diseases, including IPF. It not only involves the accumulation of excess extracellular matrix components but also likely shares intrinsic mechanisms through which structural cells activate the mesenchymal compartment by creating a fibrotic niche (1). Healthy type 2 alveolar epithelial (AT2) cells play central roles in maintaining lung homeostasis, including secreting surfactant, transporting sodium and fluids, and performing immunomodulation as well as serving as adult tissue stem cells (2) in lung tissue maintenance, repair, and regeneration. The concept that AT2 cell failure contributes to pulmonary fibrosis has been tested in multiple animal studies (3), with persistent injury signals from AT2 cells leading to elaboration of profibrogenic mediators that activate a pathologic mesenchymal response. To date, tremendous progress has been made in understanding the mechanisms underlying epithelial cell injury (4) and fibroblast activation (5) in lung fibrosis. However, challenges remain in fully understanding AT2 cell plasticity in driving lung fibrosis and intrinsic and extrinsic mechanisms leading to AT2 cell failure.

Recent findings (6–9) emphasize an important concept that healthy epithelial cells engage in continuous interactions with neighboring mesenchyme to maintain homeostasis. Moreover, the loss of these homeostatic interactions in fibrotic diseases such as IPF has only begun to be studied. Recently, single-cell RNA-Seq (scRNA-Seq) identified loss of normal epithelial cell identities and gain of abnormal transitional states of differentiation in IPF (10–14). Animal studies (11, 15) and lung organoid studies (15, 16) have also demonstrated that transitional cell states are linked to experimental lung fibrosis. Such transitional cell states are likely a common property of AT2 and other epithelial cells in the development of lung fibrosis and other chronic lung diseases. However, it remains unclear how AT2 cells lose their identities and to what extent such mechanisms contribute to fibrosis initiation and progression.

The biology of CEBPA has been studied for over 20 years. CEBPA is known to play critical roles in cellular proliferation (17), lipogenesis (18–20), lung development (21, 22), tumor suppression (23), and epithelial cell homeostasis (24). Given the significant roles of CEBPA in liver fibrosis (25), COPD, and lung fibrosis (26), we aim to understand in depth how the loss of CEBPA contributes to the depletion of AT2 cells and the promotion of lung fibrosis. Using publicly available scRNA-Seq data from patients with IPF, we confirmed that *CEBPA* is among the top downregulated genes in alveolar epithelial cells from the lungs of patients with IPF, highlighting a potentially important role of CEBPA in maintaining lung homeostasis. Therefore, we aimed to investigate the connections between loss of CEBPA expression in human IPF and its roles in the mouse models, with the goal of validating CEBPA as a valuable therapeutic target for restoring epithelial identity and fibrosis resolution.

Specifically, we took advantage of an aged mouse injury model to confirm low Cebpa expression in experimental lung fibrosis. We then tested whether deleting *Cebpa* leads to spontaneous lung fibrosis, as well as whether its loss disrupts fibrosis resolution after bleomycin exposure. Using scRNA-Seq, we obtained a better understanding of how *Cebpa* deletion disrupts tissue homeostasis. Lineage-tracing mouse and human lung organoid experiments confirmed the role of CEBPA in maintaining AT2 cell identity and tissue homeostasis, as well as initiating and resolving fibrosis. We then employed pharmacological intervention, AAV-mediated overexpression, and nongenome editing CRISPR gene activation to demonstrate the benefit of targeting CEBPA and downstream pathways in alleviating experimental lung fibrosis, supporting CEBPA as a key regulator of tissue homeostasis and repair in the lung.

## Results

### CEBPA is downregulated in human IPF lung samples.

**Figure 1:**
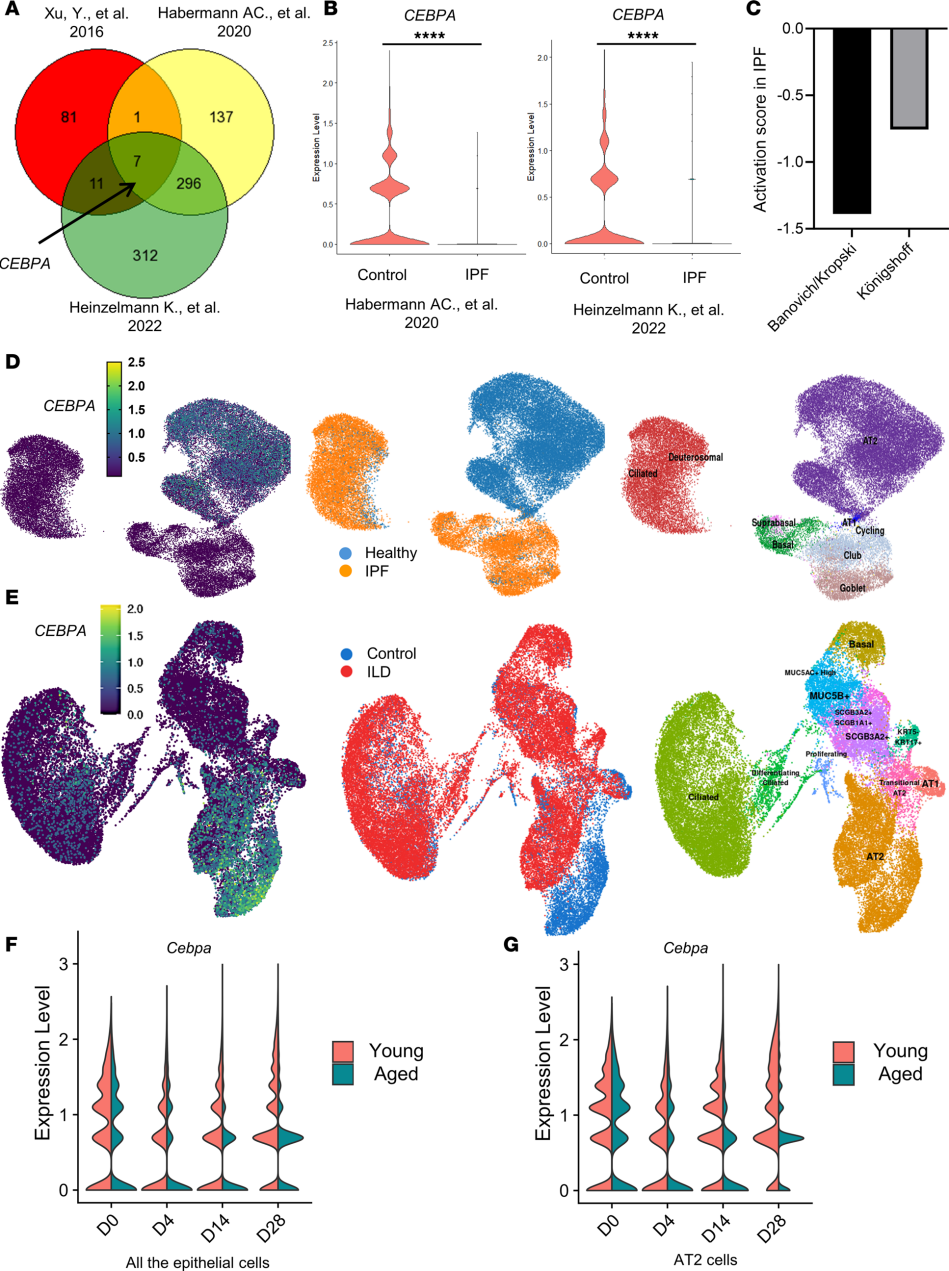
CEBPA is downregulated in human IPF and in experimental lung fibrosis. (**A**) In this Venn diagram, each circle represents genes significantly downregulated in human IPF versus control lung samples from each of 3 independent studies. (**B**) Violin plot of *CEBPA* expression in epithelial cells from the lungs of patients with and without IPF, from 2 of the human scRNA-Seq data sets analyzed (14, 27). (**C**) Activation score of Upstream Regulator Analysis (from QIAGEN Ingenuity Pathway Analysis) for the data from the 2 human scRNA-Seq studies in **B** (14, 27). (**D** and **E**) Uniform manifold approximation projection (UMAP) plots of human lung epithelial cells from the 2 scRNA-Seq data sets generated via http://ipfcellatlas.com (**F** and **G**) Violin plot of *Cebpa* expression in the epithelial cells (**F**) or AT2 cells (**G**) at different time points (day 0 [D0], D4, D14, D28) after bleomycin injury in the lung of young and aged mice from reanalysis of GSE157995. Data were analyzed using an unpaired, 2-tailed Student’s *t* test. ****P < 0.0001.

We began by querying 3 publicly available scRNA-Seq data sets to identify genes significantly downregulated in lung samples from patients with and without IPF. *CEBPA* was the most consistently downregulated gene in IPF epithelial cells (Figure 1A). *CEBPA* expression levels were significantly lower in IPF versus control epithelial cells in all available data sets (10, 14, 27) (Figure 1B). In addition, an upstream regulator analysis based on differentially expressed genes from 2 of these data sets predicted that CEBPA is negatively regulated in IPF (Figure 1C). Uniform Manifold Approximation and Projection (UMAP) representation of epithelial cells derived from available scRNA-Seq data sets (14, 27) demonstrated that *CEBPA* is enriched in AT2 cells and its expression is decreased in IPF lungs (Figure 1, D and E).

### Cebpa is downregulated in experimental lung fibrosis.

After establishing that *CEBPA* expression levels are consistently lower in the epithelial cells from human IPF versus control lung samples, we aimed to investigate Cebpa expression in an experimental lung fibrosis mice model. We exposed young (~2-month-old) and aged mice (~18-month-old) to bleomycin to simulate self-resolving (28) and persistent (29, 30) lung fibrosis, respectively. At baseline, the number of AT2 cells (represented by pulmonary-associated surfactant protein C–expressing [Sftpc-expressing] cells) did not differ between the young and aged mice. However, at day 30 after bleomycin injury, the number of AT2 cells in aged mice was significantly lower than in young mice (Supplemental Figure 1, A and B; supplemental material available online with this article; https://doi.org/10.1172/jci.insight.175290DS1). This finding demonstrates that AT2 cell numbers decline substantially in the bleomycin-induced persistent lung fibrosis aged mice model.

We then compared *Cebpa* expression levels in sorted epithelial cells from young and aged mice over the course of the experiment. In young mice, the majority of epithelial cells (CD326^+^) are Sftpc cells (Sftpc _GFP^+^), demonstrated by flow cytometry (Supplemental Figure 1C). *Cebpa* expression significantly but transiently declined after bleomycin injury, with gradual recovery by day 30 (Supplemental Figure 1D). By contrast, in aged mice, *Cebpa* expression remained significantly repressed at day 30 after bleomycin injury (Supplemental Figure 1D). We used immunostaining to confirm differences in lung tissue Sftpc and Cebpa expression at select time points (Supplemental Figure 1E). Strikingly, the transient loss of Cebpa expression in young mice and sustained loss in aged mice directly mirrored the transient versus sustained loss of AT2 cells and transient versus persistent fibrosis in young and aged mice. These data have also been validated by scRNA-Seq reanalysis from comparison of young and aged mice in the bleomycin-induced fibrosis model at different time points (day 0, day 4, day 14, day 28). UMAP (Supplemental Figure 1F) and violin plots have shown that Cebpa expression from both epithelial cells (Figure 1F) and AT2 cells (Figure 1G) was decreased after bleomycin injury in both young and aged mice and remained expressed to a lower degree in older mice than young mice at a later time (day 28) after bleomycin injury. These data consistently demonstrate that Cebpa is preferentially expressed in AT2 cells and that levels of both Cebpa and AT2 cells decrease in aged mice with persistent experimental lung fibrosis.

### Loss of Cebpa in AT2 cells promotes lung fibrosis.

**Figure 2:**
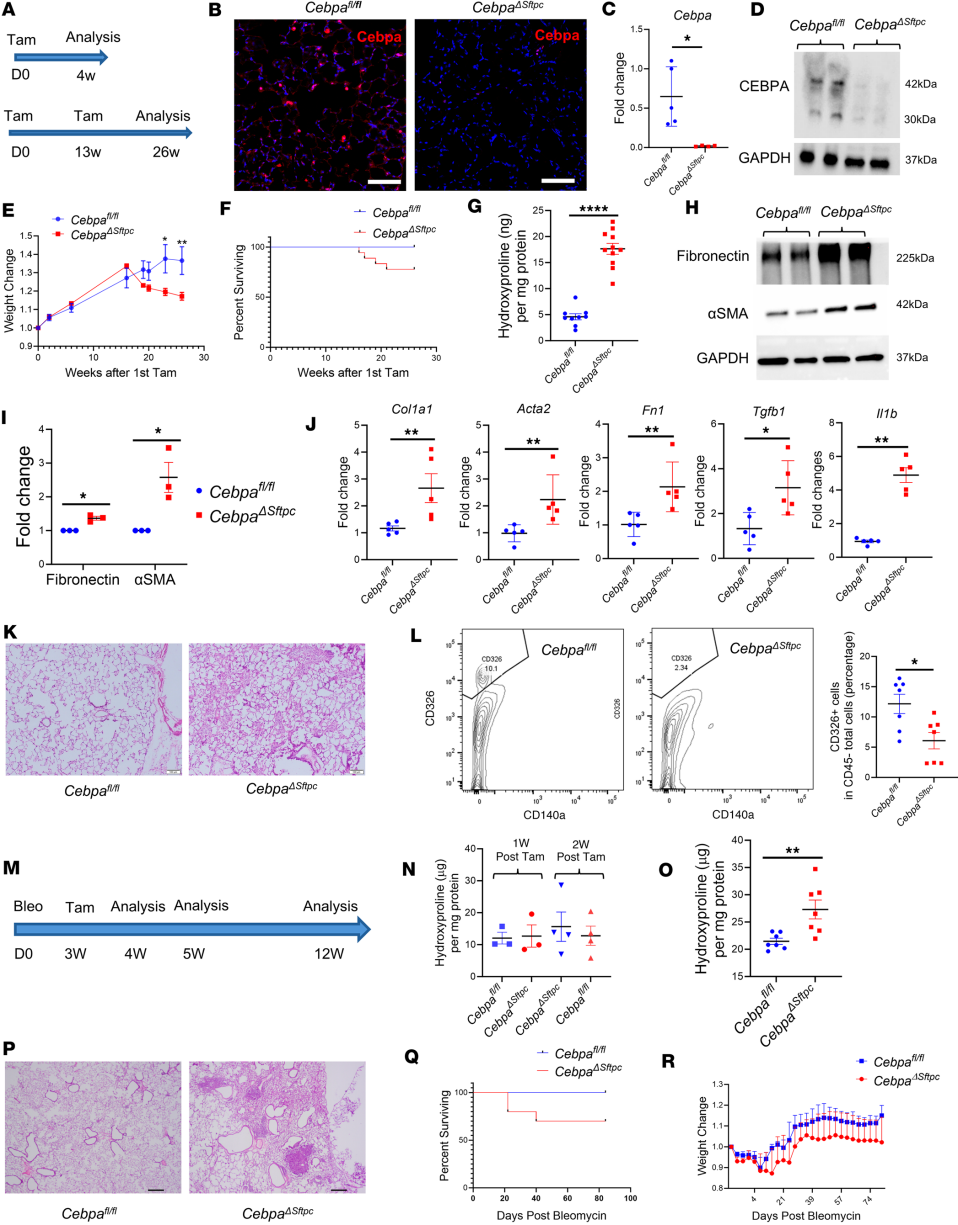
Loss of *Cebpa* in AT2 cells promotes lung fibrotic response, impairs fibrosis resolution and lung repair. (**A**) Schematic showing tamoxifen treatment and analysis timeline. (**B**–**D**) Representative immunostaining images (*n* = 3) of Cebpa expression, qPCR of *Cebpa* transcripts, and representative Western blot (*n* = 3) showing Cebpa expression from *Cebpa^ΔSftpc^* and *Cebpa^fl/fl^* mice lungs, 4 weeks after tamoxifen treatment. Scale bar: 50 μM. (**E** and **F**) Weight change of *Cebpa^ΔSftpc^* (*n* = 14) and *Cebpa^fl/fl^* (*n* = 12) mice, and survival curve of *Cebpa^ΔSftpc^* (*n* = 18) and *Cebpa^fl/fl^* (*n* = 10) mice after first tamoxifen treatment, over a 26-week period. (**G**) Hydroxyproline assay showing collagen deposition from *Cebpa^ΔSftpc^* (*n* = 11) and *Cebpa^fl/fl^* (*n* = 9) mice lungs 26 weeks after first tamoxifen treatment. (**H** and **I**) Representative Western blot and their quantification show fibronectin and α-SMA expression from *Cebpa^ΔSftpc^* and *Cebpa^fl/fl^* mice lungs 26 weeks after tamoxifen treatment. (**J**) qPCR for profibrotic gene transcripts from *Cebpa^ΔSftpc^* and *Cebpa^fl/fl^* mice lungs 26 weeks after first tamoxifen treatment. (**K**) Representative H&E staining showing lung sections from *Cebpa^ΔSftpc^* (*n* = 3) and *Cebpa^fl/fl^* (*n* = 3) mice 26 weeks after first tamoxifen treatment. Scale bar: 100 μM. (**L**) Flow cytometry analysis of CD326^+^ cells from *Cebpa^ΔSftpc^* (*n* = 7) and *Cebpa^fl/fl^* (*n* = 7) mice lungs 26 weeks after first tamoxifen treatment. (**M**) Schematic showing timeline for bleomycin treatment, tamoxifen treatment, and analysis. (**N**) Hydroxyproline assay from *Cebpa^ΔSftpc^* and *Cebpa^fl/fl^* mice lungs at 4 and 5 weeks after bleomycin injury. (**O**) Hydroxyproline assay from *Cebpa^ΔSftpc^* and *Cebpa^fl/fl^* mice lungs at 12 weeks after bleomycin injury. (**P**) Representative H&E staining showing lung sections from *Cebpa^ΔSftpc^* (*n* = 3) and *Cebpa^fl/fl^* (*n* = 3) mice at 12 weeks after bleomycin injury. Scale bar: 200 μM. (**Q** and **R**) Survival curve and weight change of *Cebpa^ΔSftpc^* (*n* = 10) and *Cebpa^fl/fl^* (*n* = 7) mice over a 12-week postbleomycin injury period. Data were analyzed using a Mann-Whitney *U* test. *P < 0.05, **P < 0.01, ****P < 0.0001.

Based on the association between low *CEBPA* levels and IPF in humans and in the mice models, we hypothesized that Cebpa loss could initiate lung fibrosis. To test this hypothesis, we generated *Cebpa*^ΔSftpc^ mice in which we conditionally deleted *Cebpa* in AT2 cells via tamoxifen-induced Cre recombinase activation (Figure 2A). At 4 weeks after tamoxifen treatment, Cebpa expression in *Cebpa*^ΔSftpc^ mice was significantly diminished compared with *Cebpa*-floxed mice (*Cebpa^fl/fl^*), as confirmed by immunostaining, quantitative PCR (qPCR), and Western blot (Figure 2, B–D). However, levels of collagen, a marker of fibrosis that can be measured with a hydroxyproline assay, were only slightly increased in the lungs of *Cebpa*^ΔSftpc^ versus *Cebpa^fl/fl^* mice (Supplemental Figure 2A), and no difference in lung tissue histology was evaluated by H&E staining and mean linear intercept measurement (Supplemental Figure 2, B and C).

To investigate the long-term effects of *Cebpa* deletion, *Cebpa*^ΔSftpc^ mice were also compared with *Cebpa^fl/fl^* mice at 26 weeks after first tamoxifen treatment (Figure 2A). *Cebpa*^ΔSftpc^ mice displayed significant weight loss (Figure 2E) and increased mortality (Figure 2F) beginning at 20 weeks after tamoxifen treatment. Hydroxyproline assay (Figure 2G) and Western blot (Figure 2, H and I) of whole lung samples demonstrated that, compared with *Cebpa^fl/fl^* mice, *Cebpa*^ΔSftpc^ mice displayed increased tissue content of total collagen, fibronectin, and α-SMA. qPCR (Figure 2J) also showed increased transcripts of the profibrotic genes *Col1a, Fn1, Acta2, Tgfb1*, and *Il1b* in lung samples from *Cebpa*^ΔSftpc^ mice. Moreover, H&E staining (Figure 2K) showed significantly increased architectural distortion in whole lung samples from *Cebpa*^ΔSftpc^ mice. Flow cytometry analysis indicated that the percentage of epithelial cells in samples from *Cebpa*^ΔSftpc^ mice also decreased (Figure 2L).

Next, we combined bleomycin-induced lung fibrosis with *Cebpa* deletion and hypothesized that mice with the *Cebpa* deletion would be particularly susceptible to developing lung fibrosis. When bleomycin was administered 4 months after tamoxifen administration (Supplemental Figure 2D), *Cebpa*^ΔSftpc^ mice lost more weight than *Cebpa^fl/fl^* mice (Supplemental Figure 2E), and half of *Cebpa*^ΔSftpc^ mice died before the 21-day endpoint (Supplemental Figure 2F). In addition, both the H&E staining (Supplemental Figure 2G) and hydroxyproline assay (Supplemental Figure 2H) revealed that *Cebpa*^ΔSftpc^ mice exhibited greater architectural distortion and higher total collagen content than *Cebpa^fl/fl^* mice.

Then, to determine if Cebpa plays a role in resolving lung fibrosis, we reversed the order of treatments, deleting *Cebpa* (via tamoxifen administration) 3 weeks after administering a bleomycin injury to initiate lung fibrosis (Figure 2M). At 4 and 5 weeks after bleomycin injury (1 and 2 weeks after tamoxifen treatment), the hydroxyproline assay did not show an obvious difference in collagen deposition between *Cebpa*^ΔSftpc^ and *Cebpa^fl/fl^* mice (Figure 2N). However, 12 weeks after bleomycin injury, we observed increased collagen deposition and more fibrotic patches (Figure 2, O and P), as well as increased mortality (Figure 2Q) in *Cebpa*^ΔSftpc^ mice, but without significant weight differences (Figure 2R). This indicates that conditional deletion of *Cebpa* after bleomycin injury compromised the ability to resolve the fibrosis initiated by bleomycin injury and restore homeostasis to the same extent as *Cebpa^fl/fl^* mice. Taken together, these data demonstrate that *Cebpa* loss in AT2 cells promotes lung fibrogenesis and impairs fibrosis resolution in mouse models.

### Single-cell transcriptome analysis reveals that Cebpa loss disrupts lung tissue homeostasis.

**Figure 3:**
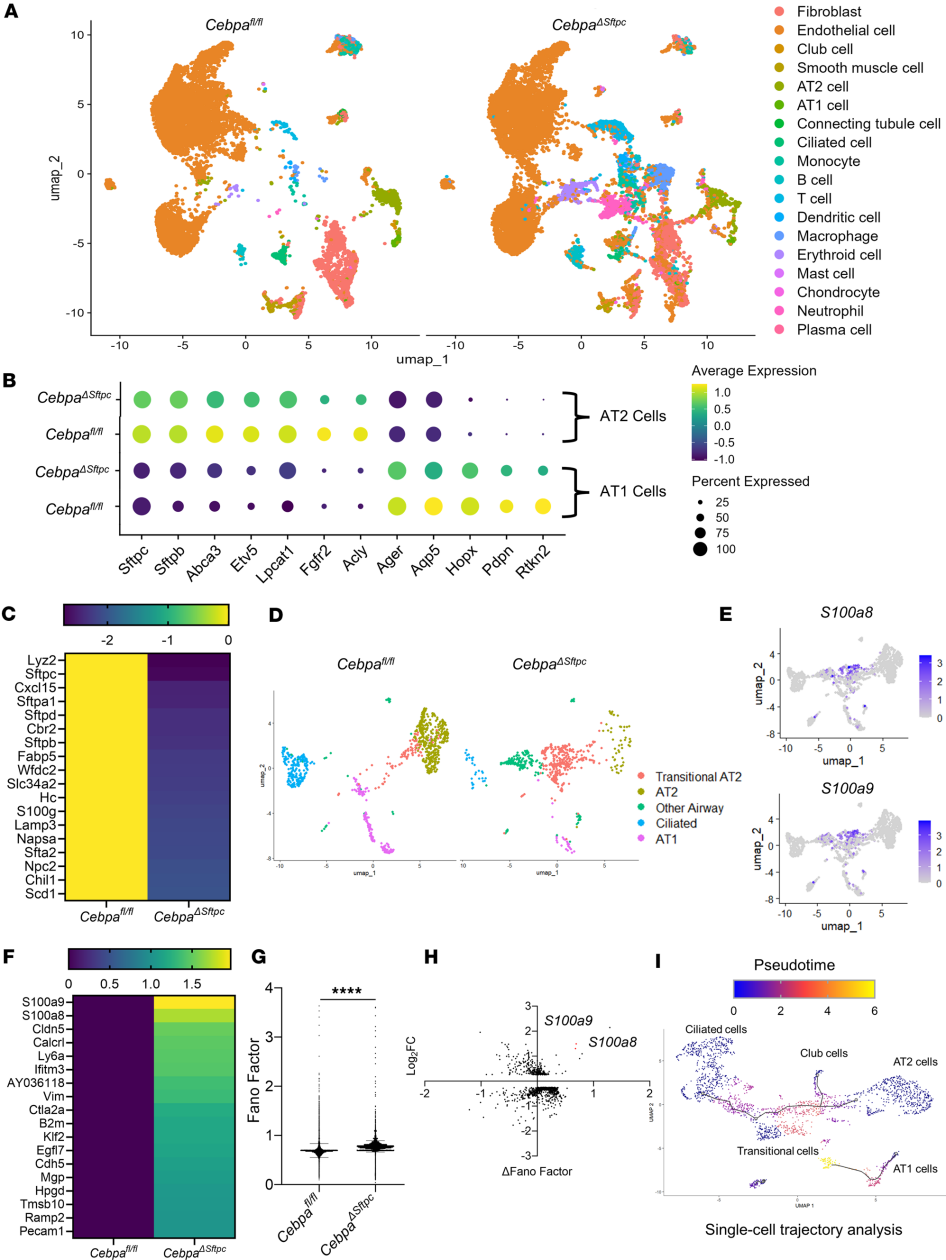
Single-cell transcriptome analysis reveals disrupted epithelial homeostasis. (**A**) UMAP plot of lung samples from *Cebpa*^ΔSftpc^ (*n* = 3) and *Cebpa^fl/fl^* (*n* = 3) mice showing major cellular clusters, each representing a different cell type. (**B**) Dot plot for AT1 and AT2 cells obtained from *Cebpa*^ΔSftpc^ versus *Cebpa^fl/fl^* mice showing (a) percentage of cells expressing a marker gene, proportional to dot size, and (b) average expression level of that gene based on unique molecular identifier (UMI) counts, accounting for differences in total UMI counts and cell-type frequencies. (**C**) Heatmap showing top downregulated genes in AT2 cells from *Cebpa*^ΔSftpc^ versus *Cebpa^fl/fl^* mice. (**D**) UMAP plot of lung epithelial cells from *Cebpa*^ΔSftpc^ versus *Cebpa^fl/fl^* mice. (**E**) UMAP plot of S100a8/a9 expression in the lung epithelial cells. (**F**) Heatmap showing top upregulated genes in AT2 cells from *Cebpa*^ΔSftpc^ versus *Cebpa^fl/fl^* mice. (**G**) Graph of transcriptional noise (represented by the Fano factor) in epithelial cells from *Cebpa*^ΔSftpc^ versus *Cebpa^fl/fl^* mice. (**H**) Transcriptional noise plotted against log_2_ fold change for genes differentially expressed between *Cebpa*^ΔSftpc^ and *Cebpa^fl/fl^* mouse epithelial cells. (**I**) UMAP plot of pseudotime single-cell trajectory analysis in epithelial cells from *Cebpa*^ΔSftpc^ versus *Cebpa^fl/fl^* mice. Data were analyzed using an unpaired, 2-tailed Student’s *t* test. ****P < 0.0001.

To better understand how loss of *Cebpa* in the AT2 cells contributes to fibrosis progression, single-cell suspensions of whole lungs were generated for scRNA-Seq from *Cebpa*^ΔSftpc^ (*n* = 3) and *Cebpa^fl/fl^* (*n* = 3) mice, which are 6 months after the first tamoxifen treatment. After filtering, normalization, and quality control, a total of 25,094 cells (11,721 *Cebpa^fl/fl^* cells and 13,373 *Cebpa*^ΔSftpc^ cells) were used for downstream analysis. Unsupervised clustering analysis revealed 18 distinct clusters in both *Cebpa*^ΔSftpc^ and *Cebpa^fl/fl^* mice, including all major known epithelial, mesenchymal, and leukocyte lineages (Figure 3A and Supplemental Figure 3A). However, significantly more immune cells were present in *Cebpa*^ΔSftpc^ mice, including neutrophils, erythroid cells, macrophages, B cells, T cells, and DCs. In contrast, there were fewer epithelial and endothelia cells in *Cebpa*^ΔSftpc^ mice (Figure 3A and Supplemental Figure 3B), suggesting the presence of inflammation and impaired tissue homeostasis in the *Cebpa*^ΔSftpc^ mice.

Markers and cell numbers for both AT2 and AT1 cells were significantly lower in *Cebpa*^ΔSftpc^ versus *Cebpa^fl/fl^* mice (Figure 3B). Pathway analysis (Supplemental Figure 3C) and heatmap analysis (Figure 3C) indicated that the most downregulated genes in AT2 cells in *Cebpa*^ΔSftpc^ versus *Cebpa^fl/fl^* mice were linked to lipid metabolism, surfactant homeostasis, and inflammation. Unsupervised clustering analysis of all the epithelial cells from the lungs of *Cebpa*^ΔSftpc^ mice revealed fewer normal epithelial cells — including AT2 cells, AT1 cells, ciliated cells, and other airway cells — than in *Cebpa^fl/fl^* mice (Figure 3D). In *Cebpa*^ΔSftpc^ mice, a unique population of transitional AT2 cells emerged, characterized by high expression of *S100a8/a9* but lower expression of *Sftpc* compared with *Cebpa^fl/fl^* mice (Figure 3E and Supplemental Figure 3D). In addition, *S100a8/9* were the genes with the greatest overexpression in AT2 cells from the lungs of *Cebpa*^ΔSftpc^ versus *Cebpa^fl/fl^* mice (Figure 3F).

Next, we quantified transcriptional noise in single cells following previous work (31, 32) as an indicator of the variability in gene expression. Transcriptional noise is often associated with loss of transcriptional control with aging, leading to a loss of cell identity. Here we observed an increase in transcriptional noise in epithelial cells from the lungs of *Cebpa*^ΔSftpc^ versus *Cebpa^fl/fl^* mice (Figure 3G). More specifically, both noise and expression levels for the *S100a8/a9* increased in samples from *Cebpa*^ΔSftpc^ mice (Figure 3H). Pseudotime trajectory analysis revealed that AT2 cells tended to become less distinct and more transitional following *Cebpa* KO (Figure 3I), suggesting that Cebpa deficiency causes a loss of epithelial identity, consistent with other studies (11, 13, 15).

### Cebpa is essential to maintain AT2 cells.

**Figure 4:**
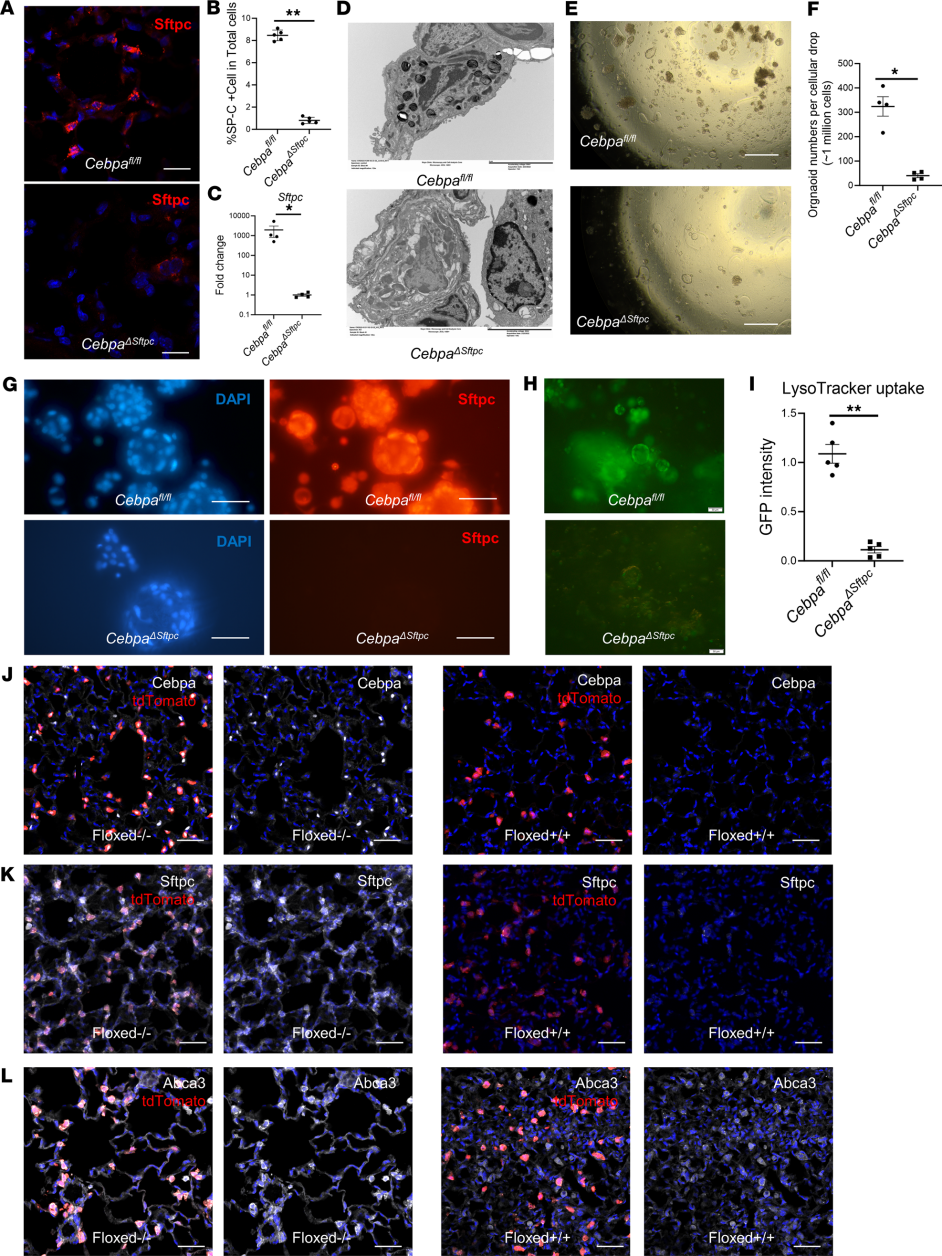
Loss of *Cebpa* induces loss of AT2 cell identity. (**A**) Representative images of immunostaining of Sftpc, a surfactant and marker of AT2 cells, in lung samples taken from *Cebpa*^ΔSftpc^ (*n* = 5) and *Cebpa^fl/fl^* (*n* = 5) mice. Scale bar: 20 μM. (**B**) Quantification of Sftpc^+^ cells as a percentage of total cells from *Cebpa*^ΔSftpc^ (*n* = 5) and *Cebpa^fl/fl^* (*n* = 5) mice. (**C**) qPCR for *Sftpc* transcripts from *Cebpa*^ΔSftpc^ (*n* = 4) and *Cebpa^fl/fl^* mice (*n* = 4). (**D**) Representative transmission electron microscopy images showing lamellar bodies, a defining feature of AT2 cells, in lung samples from *Cebpa*^ΔSftpc^ (*n* = 4) and *Cebpa^fl/fl^* (*n* = 4) mice. Scale bar: 5 μM. (**E**) Representative bright-field images of lung organoid formation from samples taken from *Cebpa*^ΔSftpc^ (*n* = 4) and *Cebpa^fl/fl^* (*n* = 4) mice. Scale bar: 200 μM. (**F**) Quantification of organoid formation from *Cebpa*^ΔSftpc^ and *Cebpa^fl/fl^* mice in **E**. (**G**) Representative immunostaining images showing Sftpc expression in the lung organoids from *Cebpa*^ΔSftpc^ and *Cebpa^fl/fl^* mice. Scale bar: 50 μM. (**H**) Representative immunofluorescence images showing LysoTracker uptake in lung organoids from *Cebpa*^ΔSftpc^ and *Cebpa^fl/fl^* mice. Organoids were stained with LysoTracker, which selectively accumulates in lamellar bodies in AT2 cells. Scale bar: 50 μM. (**I**) Quantification of LysoTracker uptake (i.e., GFP intensity) from **H**. (**J**–**L**) Representative immunostaining images of Cebpa, Sftpc, and Abca3 in lung samples from tdTomato *Sftpc* Cre lineage-tracing mice with *Cebpa^fi+/fl+^* (*n* = 3) and *Cebpa*^fl–/fl–^ (*n* = 3) mice. Each protein of interest fluoresces white, while lineage-tracing tdTomato cells fluoresce red. Scale bar: 50 μm. Data were analyzed using a Mann–Whitney *U* test. *P < 0.05, **P < 0.01.

We have shown that, in mouse models, *Cebpa* loss in AT2 cells promotes lung fibrosis and impairs its resolution, while also reducing AT2 cell–associated transcripts. Thus, we hypothesized that Cebpa is critical for maintaining AT2 cell identity. To test this hypothesis, we performed immunostaining and gene expression analyses 6 months after *Cebpa* deletion in *Cebpa*^ΔSftpc^ mice, a time point associated with significant fibrosis. Sftpc expression and the number of Sftpc^+^ cells were significantly reduced in *Cebpa*^ΔSftpc^ versus *Cebpa^fl/fl^* mice (Figure 4, A and B), indicating a loss of AT2 cells in *Cebpa*^ΔSftpc^ *mice*. qPCR (Figure 4C) also showed significantly decreased transcripts of *Sftpc* from *Cebpa*^ΔSftpc^ versus *Cebpa^fl/fl^* mice. Transmission electron microscopy confirmed that deleting *Cebpa* in AT2 cells led to loss of lamellar bodies, a defining feature of AT2 cells (Figure 4D).

To determine whether *Cebpa* plays a role in AT2 cell stemness, we created organoids from lung cells gathered from *Cebpa^fl/fl^* and *Cebpa*^ΔSftpc^ mice 6 months after *Cebpa* deletion, according to established protocols (33, 34). Organoid formation requires stemness potential of the AT2 cells, as does lung regeneration. *Cebpa* deletion in AT2 cells significantly reduced organoid formation (Figure 4, E and F), indicating that stemness potential in the lung requires *Cebpa*. Immunostaining confirmed abundant expression of Sftpc in *Cebpa^fl/fl^* organoids compared with *Cebpa*^ΔSftpc^ organoids (Figure 4G). Next, we investigated LysoTracker uptake to determine how many mature AT2 cells were present. We observed significantly reduced uptake of LysoTracker, which stains acidic compartments in live cells and selectively accumulates in lamellar bodies (35), in *Cebpa*^ΔSftpc^ versus *Cebpa^fl/fl^* organoids (Figure 4, H and I), indicting that fewer mature AT2 cells are present in the *Cebpa*^ΔSftpc^ organoids.

To further evaluate the role of Cebpa in determining the identity of AT2 cells, we generated lineage-tracing mice (tdTomato^+/+^ Sftpc-Cre^+/+^) to track the fate of AT2 cells in the presence (Cebpa-floxed^–/–^ [*Cebpa*^fl-/fl-^]) and absence of *Cebpa* (Cebpa-floxed^+/+^ [*Cebpa*^fl+/fl+^]). We hypothesized that mice with a *Cebpa* deletion (6 months after first tamoxifen) would disrupt the intact AT2 identity of those lineage-tracing tdTomato^+^ cells in their lungs. Immunostaining showed significantly reduced expression of Cebpa, Sftpc, and another AT2 cell marker, ATP Binding Cassette Subfamily A Member 3 (Abca3), in tdTomato^+^ cells from *Cebpa*-deleted mice (Figure 4, J–L). Taken together, our results show that (a) Cebpa is essential to maintain AT2 cell identity and function and (b) its deletion leads to lung fibrosis. These findings align with those from previous studies (12, 36) showing that AT2 cell dysfunction contributes to lung fibrosis initiation and progression.

### S100A8/A9 inhibitor paquinimod alleviates lung fibrosis.

Chronic inflammation prevents AT2-AT1 cell differentiation, leading to the accumulation of aberrant AT2 cells and impaired alveolar regeneration (15). Thus, we hypothesized that creating a fibrotic environment for lung organoids would decrease markers of healthy, mature AT2 cells (such as Cebpa). When we treated lung organoids from Sftpc-GFP mice with recombinant proteins S100A8/A9, *Cebpa* and *Sftpc* expression were reduced (Supplemental Figure 4), similar to effects observed under treatment with TGF-β1 (Supplemental Figure 4).

**Figure 5:**
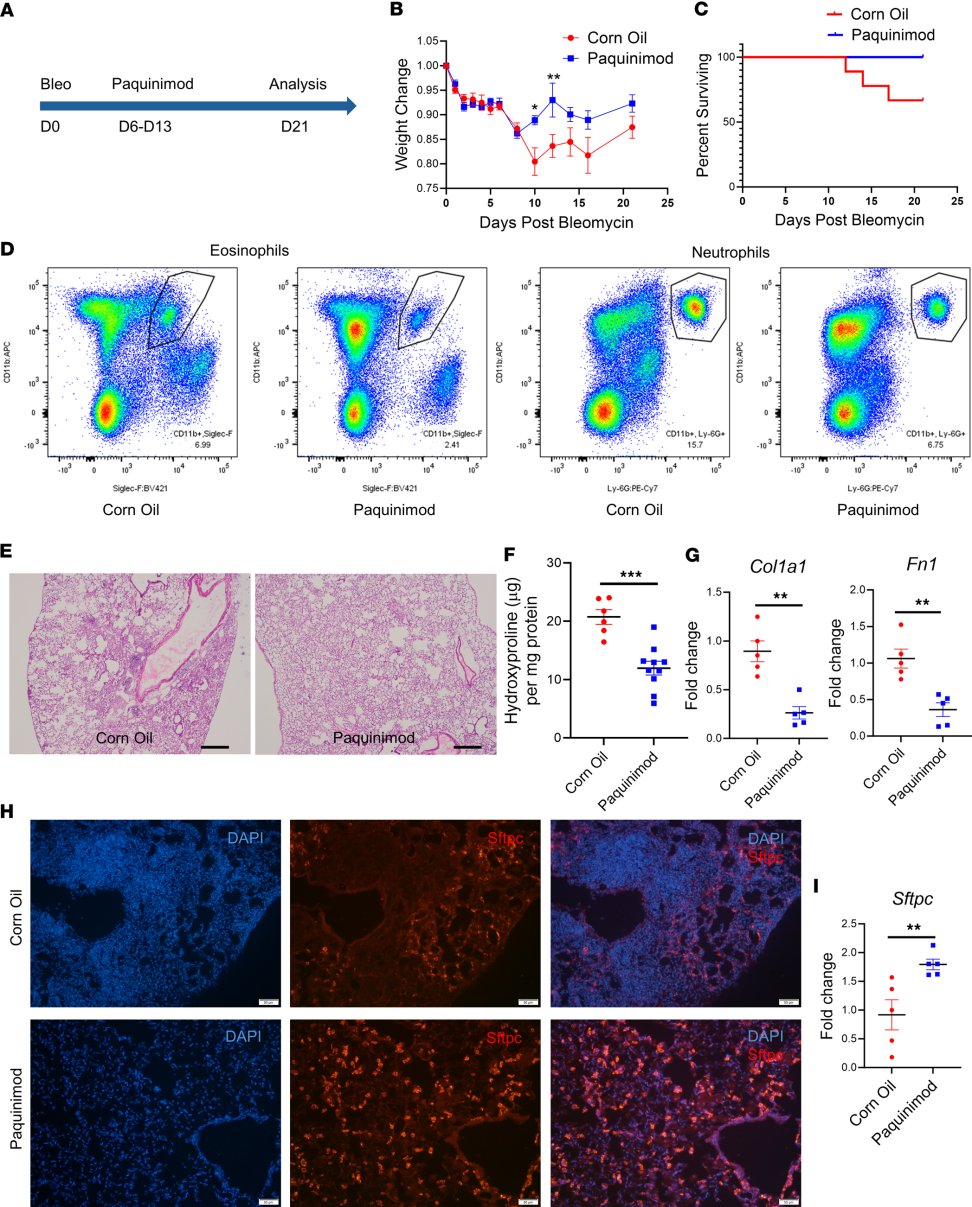
S100A8/9 inhibitor paquinimod alleviates experimental lung fibrosis. (**A**) Schematic showing timeline of paquinimod treatment, which inhibits the inflammatory S100A8/9 proteins, in the *Cebpa*^ΔSftpc^ mouse model of fibrosis. (**B**) Weight of mice treated with paquinimod (*n* = 10) or corn oil (*n* = 9), as depicted in **A**. (**C**) Survival curve for mice from **B**. (**D**) Flow cytometry analysis performed 21 days after bleomycin injury, showing eosinophils and neutrophils from the lungs of mice treated with paquinimod (*n* = 3) or corn oil (*n* = 3). (**E**) Representative H&E staining images showing lung sections taken 21 days after bleomycin injury from mice treated with paquinimod (*n* = 3) or corn oil (*n* = 3). (**F**) Hydroxyproline assay performed 21 days after bleomycin injury in lung samples from mice in **E**. (**G**) qPCR performed 21 days after bleomycin injury for profibrotic gene transcripts in mice treated with paquinimod (*n* = 5) or corn oil (*n* = 5). (**H**) Representative immunostaining images of Sftpc expression from the lungs of mice harvested 21 days after bleomycin injury and after treatment with paquinimod (*n* = 3) or corn oil (*n* = 3). (**I**) qPCR for *Sftpc* expression performed 21 days after bleomycin injury in mice from **H**. Data were analyzed using a Mann–Whitney *U* test. *P < 0.05, **P < 0.01, ***P < 0.001.

Persistent inflammation can also trigger fibrosis (37). In the previous experiments, we showed that (a) *Cebpa* loss induces persistent inflammation with prominent S100A8/A9 expression and (b) the S100A8/A9 proteins themselves can contribute to *Cebpa* repression. Therefore, we hypothesized that S100A8/A9 are mediators of fibrosis. To test our hypothesis, we employed paquinimod, an orally active quinoline-3-carboxamide class immunomodulator that targets S100A8/A9 via direct binding, blocking their interaction with their receptors (38). Paquinimod has been found to be effective in other nonlung fibrosis conditions (39–41). We treated 68-week-old aged mice i.p. with paquinimod (10 mg/kg) starting 6 days after bleomycin injection (daily for 7 days) and then harvested the mice 21 days after bleomycin injury (Figure 5A). This scheme allowed us to determine the effect of blocking S100A8/A9 in a model of persistent lung fibrosis. After 4 days, weight significantly improved in paquinimod-treated versus control mice (Figure 5B). Survival was also higher in paquinimod-treated mice (Figure 5C). Flow cytometry analysis showed that paquinimod treatment reduced neutrophil (CD45^+^CD11b^+^Ly6G^+^) and eosinophil (CD45^+^CD11b^+^Siglec-F^+^) accumulation in the lungs (Figure 5D). Histologically, paquinimod-treated mice exhibited less lung fibrosis and total collagen content than control mice, as assessed by H&E staining (Figure 5E) and the hydroxyproline assay (Figure 5F). Finally, paquinimod treatment ameliorated the increase in profibrotic gene expression (Figure 5G) and the decrease in AT2 cells (shown by Sftpc expression) observed in control mice (Figure 5, H and I). These results indicate that blocking the inflammatory signals from S100A8/A9 can alleviate lung fibrosis in a mouse model.

### Restoring Cebpa expression alleviates lung fibrosis.

**Figure 6:**
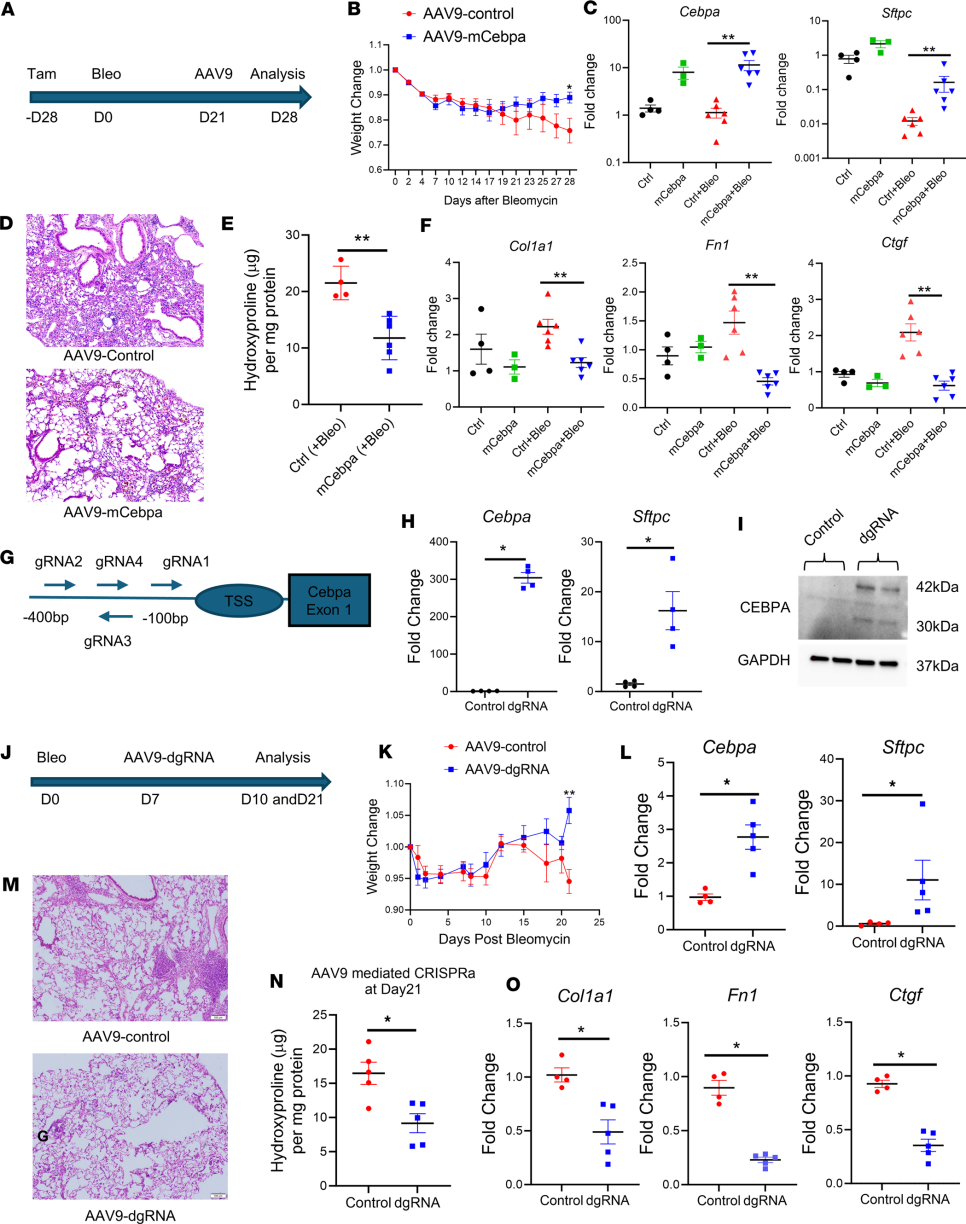
Rescue of Cebpa expression alleviates lung fibrosis. (**A**) Schematic timeline of AAV9 treatment to alleviate lung fibrosis. (**B**) Weight of *Cebpa^ΔSftpc^* mice treated with AAV9-mCebpa (*n* = 6) or AAV9-control (*n* = 5). (**C**) qPCR for Cebpa and *Sftpc* transcripts performed 28 days after bleomycin injury on sorted epithelial cells from *Cebpa^ΔSftpc^* mice lungs treated with AAV9-mCebpa or AAV9-control. (**D**) Representative H&E staining showing 28 days after bleomycin injury from *Cebpa^ΔSftpc^* mice lung treated with AAV9-dgRNA (*n* = 3) or AAV9-control (*n* = 3). (**E**) Hydroxyproline assay performed 28 days after bleomycin injury from *Cebpa^ΔSftpc^* mice lungs treated with AAV9-mCebpa (*n* = 6) or AAV9-control (*n* = 5). (**F**) qPCR for profibrotic gene transcripts performed 28 days after bleomycin injury from *Cebpa^ΔSftpc^* mice lungs treated with AAV9-mCebpa or AAV9-control. (**G**) Schematic showing how endogenous *Cebpa* expression was enhanced using an inactivated CRISPR-Cas9 system. (**H**) qPCR for *Cebpa* and *Sftpc* transcripts in cultured epithelial cells from Cas9 mice and then transfected with the dgRNA1 plasmid (*n* = 4). (**I**) Representative Western blot showing *Cebpa* activation in cultured epithelial cells from Cas9 mice treated with the dgRNA1 plasmid (*n* = 3). (**J**) Schematic timeline showing endogenous *Cebpa* reactivation in the 52- to 60-week-old Cas9 mice with bleomycin induced lung fibrosis. (**K**) Weight change of Cas9 mice after bleomycin injury and after treatment with AAV9-dgRNA (*n* = 5) or AAV9-control (*n* = 5). (**L**) qPCR of *Cebpa* and *Sftpc* transcripts performed 10 days after bleomycin injury on sorted epithelial cells from Cas9 mice treated with AAV9-dgRNA or AAV9-control. (**M** and **N**) Representative H&E staining (*n* = 3) and hydroxyproline assay showing 21 days after bleomycin injury from Cas9 mice lungs treated with AAV9-dgRNA or AAV9-control. (**O**) qPCR for profibrotic gene transcripts performed 21 days after bleomycin injury from Cas9 mice lungs treated with AAV9-dgRNA (*n* = 5) or AAV9-control (*n* = 4). Data were analyzed using a Mann-Whitney *U* test. *P < 0.05, **P < 0.01.

Because *Cebpa* KO induces long-lasting lung fibrosis, we aimed to determine if restoring Cebpa could alleviate lung fibrosis. First, we administered tamoxifen to delete *Cebpa* in *Cebpa*^ΔSftpc^ mice, and 4 weeks later, we administered a single dose of bleomycin intratracheally to induce experimental lung fibrosis. Twenty-one days after bleomycin injury, mice received a single intratracheal dose of 5 × 10^10^ genome copies of AAV9-mCebpa or AAV9-control. We then performed analyses 28 days after bleomycin injury (Figure 6A). After day 21 after bleomycin injury, mice treated with AAV9-mCebpa (those with restored Cebpa expression) exhibited less weight loss than control mice (Figure 6B). At 28 days after bleomycin injury, *Sftpc* and *Cebpa* expression from sorted lung epithelial cells was restored by AAV9-mCebpa treatment, both with and without bleomycin treatment (Figure 6C). Architectural distortion (Figure 6D) and total collagen in the lungs of mice treated with AAV9-mCebpa was also significantly lower (Figure 6E). Bleomycin increased profibrotic gene transcripts in whole lung tissue from control mice, but this increase was attenuated by treatment with AAV9-mCebpa (Figure 6F).

To explore the relevance of targeting Cebpa to treat human lung fibrosis, we generated organoids from human lung tissue (Supplemental Figure 5A) and treated them with TGF-β1 (10 ng/mL) to induce a fibrotic phenotype with or without *Cebpa* overexpression (Supplemental Figure 5B). Overexpression of *Cebpa* significantly upregulated *Sftpc* expression in the lung organoids derived from sorted mouse lung epithelial cells (Supplemental Figure 5C). For human lung organoids, representative bright-field images showed mixed cellular populations, including epithelial spheres and fibroblasts present in the lung organoids (Supplemental Figure 5D). The goal of using mixed cell culture is to recapitulate the effects of Cebpa gene delivery in vivo, since AAV might infect both epithelial cells and fibroblasts in the lung. Delivery of Cebpa to these human lung organoids via AAV9-mCebpa treatment increased *SFTPC* expression and decreased profibrotic gene expression (Supplemental Figure 5E), which help us understand the overall effects of this approach, specifically the restoration of AT2 gene expression and antifibrotic effects simultaneously.

We also tested the ability to enhance endogenous *Cebpa* expression using an inactivated CRISPR-Cas9 system. In this system, sgRNAs target sequences within −400 and +100 bp of the transcriptional start site (TSS, in particular between −100 and +50 bp) to enhance transcriptional activation (42–44). Following the protocol published by Liao et al. (45), we created an AAV-dgRNA_Cebpa construct and identified 4 target sequences near the *Cebpa* TSS (Figure 6G), which were inserted into plasmids. To test the effectiveness of these 4 gRNAs, we cultured sorted lung CD326^+^ cells from Cas9 mice and transfected them with the 4 individual AAV-dgRNA_Cebpa plasmids in vitro and compared their *Cebpa* expression (Supplemental Figure 6). The gRNA1 plasmid significantly increased the expression of Cebpa (Figure 6, H and I) as well as *Sftpc* expression (Figure 6H). To study the effect of endogenous *Cebpa* reactivation in a mouse model of fibrosis, a single dose of 5 × 10^11^ genomic copies of AAV9-dgRNA_Cebpa or AAV9-control was injected intratracheally into the lungs of Cas9 mice 7 days after administering bleomycin (Figure 6J). Mice that received AAV9-dgRNA_Cebpa lost less weight than control mice at day 21 (Figure 6K). At day 10 after bleomycin injury, AAV9-dgRNA_Cebpa treatment significantly upregulated both *Cebpa* and *Sftpc* expression in sorted epithelial cells (Figure 6L). At day 21 after bleomycin injury, H&E staining (Figure 6M) and the hydroxyproline assay (Figure 6N) further demonstrated that treatment with AAV9-dgRNA_Cebpa versus the control vector reduced total collagen content. AAV9-dgRNA_Cebpa treatment also reduced profibrotic gene expression in whole-lung tissue samples (Figure 6O). These results indicate that restoring endogenous Cebpa expression in the lung is able to accelerate fibrosis resolution and alleviates fibrotic response in a mouse model as well as a human lung organoid model of fibrosis.

## Discussion

In this study, we set out to identify a candidate gene involved in fibrosis, confirm its contribution to the disease process, and investigate the molecular mechanisms responsible. By comparing unbiased gene expression of epithelial cell populations from human IPF versus control lung samples, we identified loss of CEBPA as a candidate regulator of epithelial dysfunction in AT2 cells from IPF samples. Next, we used mouse models of lung fibrosis to establish Cebpa’s role in epithelial identity, tissue homeostasis, and repair in the lung. Deleting Cebpa in AT2 cells was sufficient to initiate mild, spontaneous fibrosis in mice, and deletion significantly amplified and prolonged the fibrotic response to bleomycin injury. *Cebpa* KO in mouse AT2 cells also resulted in the loss of surfactant proteins and lamellar bodies and increased inflammation levels, demonstrating that Cebpa deficiency causes AT2 cells to lose key markers of their identity. Lastly, restoring *Cebpa* expression via AAV-mediated gene overexpression or CRISPR activation was sufficient to protect AT2 cells and diminish lung fibrosis in mouse models, as well as human lung organoids.

These findings are consistent with a growing body of literature showing epithelial alterations in scRNA-Seq studies from patients with IPF (10, 14, 27, 46). Recent studies have shown that transitional AT2 cells or indeterminate epithelial cells mediate pulmonary fibrosis, displaying aberrant activation of the TGF-β, ER stress, and TP53 signaling pathways (11). Our findings that *Cebpa* KO causes AT2 cell surfactant protein loss, lamellar body loss, and increased inflammation demonstrates that AT2 cells can lose their identity due to *Cebpa* deficiencies. We report that Cebpa is crucial to maintaining AT2 cell identity, which is consistent with its role in epithelial maintenance by preventing epithelial-to-mesenchymal transition (24). CEBPA has been known as a master regulator of adipogenesis, which control lipids generation (18, 19). CEBPA has been also studied mainly in the embryonic lung (21, 22). While many factors contribute to lung development and maturation, their losses do not necessarily lead to lung fibrosis. The uniqueness of lower CEBPA expression in the IPF highlights its association with fibrosis phenotype. Previous work demonstrated that specific deletion of *Cebpa* in the lung epithelium blocks the production of surfactant lipids and proteins necessary for lung function, which cause lung epithelium immaturity and respiratory failure (22). Mice that survived postnatally from embryonic deletion of *Cebpa* developed COPD and fibrosis phenotypes characterized by histology and inflammatory indicators (26). Our findings linking *Cebpa* loss to a fibrotic phenotype in the lungs of adult mice build on these earlier findings, highlighting the pathobiological relevance of *Cebpa* deficiency in the AT2 cells from human IPF lungs.

Prior studies have shown that bleomycin-induced lung fibrosis spontaneously resolves in young mice and, thus, fails to fully recapitulate IPF phenotypes in humans, including aberrant epithelial remodeling and progressive fibrosis (28, 29). Thus, there remains an unmet need for a preclinical model with features that more closely resemble the human IPF. By contrast, the persistent pulmonary fibrosis model we present in this study has strong clinical relevance, as evidenced by the *CEBPA* deficiency we documented in AT2 cells from patients with IPF. Fibrosis is not maintained by bleomycin-induced lung injury but is induced and sustained by *Cebpa* KO in AT2 cells, in agreement with the role for AT2 cell dysfunction in mediating lung fibrosis (12, 36).

It is well known that persistent inflammation increases with age, contributing to organ dysfunction and fibrosis (47). However, the molecular mechanisms linking age, inflammation, and fibrosis remain mysterious. Aging is often associated with cellular “fate drifts” and ambiguous cell-type identities (48, 49). Aging can also result in dysregulated transcriptional programs that lead to transcriptional noise, which may contribute to inflammation. Transcriptional noise serves as an indicator for the variability in gene expression and is often associated with the loss of transcriptional control, particularly with aging, leading to a loss of cell-type specificity. Aging involves stochastic changes rather than a precisely orchestrated alteration in gene activity. For example, scRNA-Seq studies have shown that lung cells (50) and pancreatic cells (49) become transcriptionally noisy with age. Increased transcriptional noise and deregulated epigenetic control were found in the lungs of aged mice. Individual endocrine cells in older individuals were more likely to express irrelevant hormone genes, mirroring our findings that the loss of Cebpa indicates a loss of transcriptional control in AT2 cells, resulting in increased expression of erratic genes such as *S100a8* and *S100a9*. Therefore, progressive fibrosis could result from transcriptional noise and erratic inflammation disrupting AT2 cells homeostasis. Consistent with this hypothesis, we showed that AT2 cells from mouse lungs harboring *Cebpa* KO displayed significantly increased transcriptional noise across lung epithelial populations, leading to disrupted tissue homeostasis and aberrant inflammation. These aberrant AT2 cells were characterized by increased expression of S100a8/a9, which are also upregulated during aging (51) and play a crucial role in fibrosis onset and the subsequent formation of a feed-forward fibrotic loop that promotes fibrosis (51–53). Furthermore, we have shown that the S100A8/A9 inhibitor paquinimod effectively alleviated fibrotic response and restored AT2 cell populations in a bleomycin-induced lung fibrosis aged mice model. We also acknowledged that the likely effects of paquinimod might not only result from regulating epithelial cells but also from blocking the proinflammatory and profibrotic effects of monocytes. This represents a limitation of this experiment. In short, our study presents a mechanism by which *Cebpa* deficiency in lung AT2 cells links aging, disrupted homeostasis, and fibrosis.

Our experiments also suggest that increasing *CEBPA* might be protective or therapeutic in IPF. However, *CEBPA* is a transcription factor that is considered to be an undruggable target. Increasing gene expression by delivery and overexpression from plasmids via AAVs is a potential strategy for targeting this undruggable pathway. We also note that intratracheal instillation of AAVs might infect multiple cell types including macrophages and fibroblasts in the lung. Here, we also used a CRISPR activation system to enhance *Cebpa*, which involves the use of a modified version of the CRISPR-Cas9 system. In CRISPR activation, a deactivated version of the Cas9 enzyme (called dCas9) is fused with transcriptional activator domains and is guided to a specific DNA sequence by a gRNA complementary to the target gene’s sequence (54). Once the dCas9-gRNA complex binds to the promoter of the target gene, the transcriptional activator domain recruits transcriptional machinery, leading to increased target gene expression. It has the advantages of bypassing the size limitation of AAV gene delivery (45), avoiding the need for genome editing/insertion (55), limiting the risk of aberrant overexpression, and ensuring a physiological range of expression levels (56) depending on basal gene expression levels and the gene’s epigenetic status. Clearly, the safe and efficient delivery of a CRISPR activation system in vivo, as performed here, remains a major challenge to the widespread clinical success of any CRISPR-Cas9 therapeutics (57). Our study provides an initial proof of concept that insufficient gene expression can be restored by CRISPR activation in vivo to alleviate a model of chronic lung disease. Future research may uncover the epigenetic mechanism of CEBPA repression in human pulmonary fibrosis, potentially leading to additional strategies for restoring gene regulation.

In conclusion, our results demonstrate that CEBPA plays a critical role in AT2 cell maintenance and tissue homeostasis in the mouse lung. We speculate that lower levels of CEBPA expression in the lungs of patients with IPF are a central feature of chronic lung injury characterized by AT2 cell loss, persistent inflammation, and disrupted tissue homeostasis. Our work provides mechanistic insights into the regulation of AT2 cell identity by *Cebpa* and by *Cebpa* deficiency–induced inflammation, characterized by increased *S100a8/a9* expression. Moreover, we have shown that restoring *Cebpa* expression was sufficient to alleviate fibrosis (Supplemental Figure 7). Future research will be necessary to uncover the epigenetic mechanism responsible for CEBPA repression in human IPF and to design a more precise gene restoration method based on that specific mechanism. In the future, such an approach could be harnessed to rescue expression of CEBPA or other regulators of aberrant epithelial cell types in patients with IPF and other chronic lung diseases, to restore tissue homeostasis and promote lung repair.

## Methods

### Sex as a biological variable.

Sex was not considered as a biological variable. Both male and female mice were included in this study.

### Human specimens.

Human lung samples were used to generate lung organoids. Lung tissues were collected from patients whose “healthy” lungs were deemed unsuitable for transplantation due to size incompatibility or were unsuitable for lung transplantation.

### Mouse models.

Sftpc-CreERT2 mice (58), *Cebpa* “floxed” allele mice (59), and tdTomato Cre mice (60) were purchased from The Jackson Laboratory. All mice analyzed had mixed genetic backgrounds and were age matched unless mentioned specifically. The mice used for experiments were 3 months old unless otherwise indicated. *Cebpa* conditional KO in the AT2 cells was conducted in the Sftpc-CreER^+^ *Cebpa^fl/fl^* mice (referred to as *Cebpa*^ΔSftpc^ mice) and their control *Cebpa^fl/fl^* mice. Additional lineage tracing was carried out in Sftpc-CreER^+^ tdTomato^+^ mice, which have tdTomato labeling with (*Cebpa^fl+/fl+^*) or without (*Cebpa^fl−/fl−^*) the deletion of Cebpa in the AT2 cells after tamoxifen treatment. Cas9 mice were purchased from The Jackson Laboratory (Rosa26-Cas9 knockin on B6J, stock no. 026179). These CRISPR/Cas9-knockin mice constitutively express CRISPR-associated protein 9 (cas9) endonuclease and EGFP in a widespread fashion under the direction of a CAG promoter, which can be used with designed gRNAs for manipulating gene expression in vivo or ex vivo.

### Mouse experimental fibrosis experiments.

*Cebpa* deletion was induced with 5 injections of 75 mg tamoxifen/kg body weight, daily. A second round of weekly tamoxifen injections was given if analyses were performed more than 3 months after the initial round of tamoxifen injection. To induce experimental fibrosis, bleomycin (0.5 U/kg in 50 μL saline) was administered to the mice intratracheally, as previously described (29). Mice were then randomized to receive 10 mg/kg/day paquinimod (or corn oil) i.p. for 7 days to inhibit the effect of S100a8/a9. Mice were also randomized to receive a single intratracheal dose of AAV9-mCebpa, AAV9-dgRNA_Cebpa, or AAV9-empty control. Lungs were collected at designated time points.

### Lung harvest, FACS, and analysis.

Mice were anaesthetized with 90–120 mg/kg ketamine combined with 10 mg/kg xylazine, after which their lungs were perfused with ice-cold PBS. The lungs were then immediately harvested, minced in 10 cm petri dishes, and incubated in digestive solution: DMEM (Thermo Fisher Scientific, 11965092), 0.2 mg/mL Liberase DL (MilliporeSigma, 5401119001), and 100 U/mL DNase I (MilliporeSigma, 4536282001). Tissue was digested at 37°C for 40 minutes. Digestive solution was inactivated with 1× volume DMEM (Thermo Fisher Scientific, 11965092) containing 10% FBS (Thermo Fisher Scientific, A5256701). Cell and tissue suspensions were subjected to a 40 μm filter and centrifuged at 300*g* for 5 minutes. Cell pellets were then resuspended in RBC lysis buffer (BioLegend, 420301) for 90 seconds and diluted in a 4× volume of PBS. Cells were centrifuged at 300*g* for 5 minutes and resuspended in 200 μL MojoSort Buffer (BioLegend, 480017). Single-cell suspensions were then incubated with PerCpCy5.5 anti–mouse CD45 (BioLegend, 1:200, 157612), PE anti–mouse CD326 (BioLegend; 1:200, 118206), and DAPI (Thermo Fisher Scientific, 1:1,000, 62248) antibodies for 30 minutes on ice. To isolate and analyze CD326^+^ cells, cells were sorted by FACS using a BD FACS Aria II (BD Biosciences). The following selection strategy was used: debris exclusion (forward scatter area [FSC-A] by side scatter A [SSC-A]), doublet exclusion (SSC width [SSC-W] by SSC height [SSC-H] and FSC-W by FSC-H), dead cell exclusion (DAPI^–^), CD45 cell removal. Epithelial cells were sorted as DAPI^–^CD45^–^CD326^+^ cells. For RNA analysis, CD326^+^ cells were sorted directly into RLT lysis buffer (Qiagen, 74004). To analyze eosinophils and neutrophils, additional antibodies for APC anti-CD11b (BioLegend, 1:200, 101211), BV421 anti-mouse CD170 (Siglec-F, BioLegend, 1:200, 155509), and PE/Cyanine7 anti–mouse Ly-6G (BioLegend, 1:200, 127617) were included in the antibody incubation. Population analysis was performed using FlowJo FACS analysis software (BD Biosciences).

### Lung organoid culture and primary cell culture.

Briefly, fresh lung tissue was cut in small pieces (1–2 mm^2^) and incubated in digestive solution (100 U/mL DNase I plus 0.2 mg/mL Liberase at 35 minutes for mouse lung, 2 mg/mL collagenase I at 90 minutes for human lung). After 70 μm filter and cells were embedded into a Matrigel (Corning, 356231) dome, which was incubated at 37°C for 30 minutes to allow time for polymerization. Once embedded, the explants were fed with AT2 medium following an established protocol (34). The medium was changed every other day. For mouse lung epithelial cells organoids, DAPI^–^CD45^–^CD326^+^ cells will be used for Matrigel culture. For monolayer epithelial cell culture, cells were cultured in Epithelial Cell Medium (Cell Biologics, M6621) with 5% CO_2_ and 95% air in a humidified incubator. Transient transfection was performed using a Lipofectamine 3000 kit (Thermo Fisher Scientific, L3000015) according to the manufacturer’s instructions.

### CRISPR activation in vitro and in vivo.

Following the protocol published by Liao et al. (45), we constructed sgRNAs that identified 4 target sequences near the *Cebpa* TSS (Figure 6G). These predesigned, 14-bp gRNA spacers (5′–3′; GCGCAGGAGTCAGT; GGGCTCCCTAGTGT; CTGCAAGGCGAACC; ACAGCGCCGCCGGG) were inserted into plasmid AAV-U6-dgRNA-CAG-MPH, which utilizes a modified version of the Synergistic Activation Mediator (SAM) system. This plasmid was originally produced by Juan Belmonte (Addgene plasmid 106259) and expresses the transcription activator MS2:P65:HSF1 (MPH) from the CAG promoter and one U6-driven dgRNA in an AAV backbone. For the in vitro CRISPR activation experiment, AAV-U6-dgRNA-CAG-MPH plasmids carrying dgRNA_Cebpa (AAV-dgRNA_Cebpa) were transiently transfected into sorted CD326^+^Cas9^+^ cells using a Lipofectamine 3000 kit (Invitrogen) according to the manufacturer’s instructions. For the in vivo CRISPR activation experiment, AAV9 plasmids expressing AAV-dgRNA_Cebpa or AAV-control were produced by the University of Pennsylvania Vector Core. AAV9 was delivered to the mouse lung intratracheally, following an established protocol for bleomycin delivery (29).

### Hydroxyproline assay for measuring collagen.

Collagen content in the lung was measured using a hydroxyproline assay kit (Abcam, ab222941) according to the manufacturer’s instructions, as previously described (61). Frozen lung tissue was homogenized in sterile water at a ratio of 10 mg tissue to 100 μL water and hydrolyzed in 12M HCl in a pressure-tight, Teflon-capped vial at 120°C for 3 hours, followed by filtration through a 45 μm Spin-X Centrifuge Tube filter (Corning, 8162). Next, a 10 μL sample was dried in a Speed-Vac overnight, followed by incubation with 100 μL Chloramine T reagent for 5 minutes at room temperature and then incubation with 100 μL DMAB for 90 minutes at 60°C. The absorbance of oxidized hydroxyproline was measured at 560 nm. Hydroxyproline concentrations were calculated from a standard curve generated using known concentrations of trans-4-hydroxyl-L-proline. The total amount of protein isolated from the weighed tissues was determined by using a protein assay kit (Bio-Rad). Hydroxyproline content data were expressed as μg collagen per mg total lung protein (μg/mg).

### RNA extraction and quantitative PCR.

Total RNA was isolated from samples using the RNeasy Plus Mini kit (Qiagen, 74134) or RNeasy Micro Kit (Qiagen, 74004) depending on the RNA quantities. cDNA was synthesized with SuperScript IV Reverse Transcriptase (Thermo Fisher Scientific, 18090050). Quantitative PCR (qPCR) amplification was carried out using the FastStart Essential DNA Green Master (Roche, 06402712001) for SYBR Green I-based real-time PCR on the Lightcycler 96 Real-Time PCR System (Roche), according to the manufacturer’s instructions. qPCR was performed by incubating plates at 95°C for 10 minutes and then cycling 40 times at 95°C for 10 seconds, 60°C for 10 seconds, and 72°C for 10 seconds. Ct values within each experiment were normalized against GAPDH/Gapdh levels, and fold change was calculated for all conditions relative to a single, randomly selected, control result. Both human and mice primers are listed in Table 1.

### Tissue preparation, H&E staining, and immunostaining.

Lungs were inflated with 4% formaldehyde solution (Thermo Fisher Scientific, 28908) and continually fixed in 4% formaldehyde at 4°C for 24 hours. Then the lungs were cryoprotected in 30% sucrose and embedded in OCT (Fisher HealthCare, 4585). H&E staining followed the standard H&E protocol. Briefly, slides were washed with water to remove the OCT. The nuclei were stained with hematoxylin (Abcam, ab150678) for 2 minutes, and the cytoplasm was stained with eosin (MilliporeSigma, HT110280) for 3 minutes. Slices were sealed with neutral resin after the dehydration and clearing steps.

Tissue sections (10 μm) from each block were cut in a cryostat at –21°C and mounted onto Fisherbrand Tissue Path Superfros Plus Gold slides (Thermo Fisher Scientific). Slides were permeabilized in 0.25 % Triton X-100 (Sigma-Aldrich, X-100), blocked with 1% BSA for 1 hour, and incubated overnight with CEBPA antibody (dilution 1:100, Cell Signaling Technology, 8178S), SFTPC antibody (dilution 1:100, Santa Cruz Animal Health, PA5-71680), or ABCA3 antibody (dilution 1:100, Thermo Fisher Scientific, PA5-103632) at 4°C overnight in PBS with 1% BSA, followed by incubation with a mixture of fluorescence-conjugated secondary antibody (dilution 1:500, Thermo Fisher Scientific) and DAPI (dilution 1:1,000, Thermo Fisher Scientific) at room temperature for 1 hour. All images were captured using a Zeiss LSM 780 confocal microscope or an Olympus CKX53 microscope.

### Western blotting.

Cells were harvested into RIPA Lysis Buffer (Thermo Fisher Scientific, 89900) with Halt Protease and Phosphatase Inhibitor Cocktail (Thermo Fisher Scientific, 78440). Lysates were then quantitated using Pierce BCA Protein Assay Kit (Thermo Fisher Scientific, 23225), and equal amounts of protein were subjected to 4%–15% Mini-PROTEAN TGX Gel (Bio-Rad, 15 μL [4568086] or 50 μL [4561084]). Proteins were transferred from the gel to PVDF membranes with Trans-Blot Turbo Transfer System (Bio-Rad). After blocking for 1 hour at room temperature with 5% nonfat dry milk (Bio-Rad, Bloting-Grade Blocker, 1706404) in TBST blocking buffer, PVDF membranes were probed with GAPDH (Cell Signaling Technology, 2218S), Cebpa (Cell Signaling Technology, 8178S), α-SMA (Sigma-Aldrich, F3777), and Fibronectin (Santa Cruz Biotechnology Inc., sc-81767) antibodies at 4°C overnight followed by incubation with HRP-conjugated goat anti-rabbit antibody (Promega, W4011) for 1 hour at room temperature. Bands were detected using Super Signal West Pico Plus (Thermo Fisher Scientific, 34580) and visualized using a Bio-Rad ChemiDoc Imaging system (Bio-Rad). Quantification was performed via densitometry. Expression of a specific antibody relative to GAPDH was computed using the ImageLab software provided by Bio-Rad.

### Transmission electron microscopy.

Lung tissue was placed into fixative containing 4% paraformaldehyde and 1% glutaraldehyde in 0.1M phosphate buffered solution, pH 7.2 (PBS). Then the fixed tissue was washed with PBS, stained with 1% osmium tetroxide, washed in H_2_O, stained with 2% uranyl acetate, washed in H_2_O, dehydrated through a graded series of ethanol and acetone, and embedded in Embed 812 resin. Following a 24-hour polymerization at 60°C, 0.1 μM ultrathin sections were prepared and poststained with lead citrate. Micrographs were acquired using a JEOL 1400 Plus transmission electron microscope (JEOL Inc.) at 80 kV equipped with a Gatan Orius camera (Gatan Inc.).

### scRNA-Seq and analysis.

In our mouse fibrosis experiments involving *Cebpa*-KO mice, 26 weeks after the first tamoxifen injections, the lungs of *Cebpa*^ΔSftpc^ (*n* = 3) and *Cebpa^fl/fl^* (*n* = 3) mice were harvested and disassociated into single-cell suspensions as described earlier. Cell suspensions were brought to the Genome Analysis Core at Mayo Clinic for processing. Cell viability and counts were determined using a Countess II Automated Cell Counter. Single cells, reagents, and a single Gel Bead containing barcoded oligonucleotides were encapsulated into a nanoliter-sized Gel Bead-in-Emulsion using the 10X Genomics GemCode platform (10X Genomics). cDNA libraries were prepared with a Chromium Single Cell 3′ GEM Library & Gel Bead Kit (10X Genomics). All cDNA libraries were sequenced on an Illumina HiSeq 4000 instrument.

scRNA-Seq data were aligned and quantified using the 10X Genomics Cell Ranger Software Suite (v6.1.1) against the mouse reference genome mm10 from raw count data. Data were processed and analyzed with the Seurat R package V4.0 (62) to perform integrated analyses of single cells. Genes expressed in < 3 cells and cells that expressed < 200 genes and > 20% mitochondria genes were excluded from downstream analysis in each sample. One Seurat object was created from filtered count matrices from pooled *Cebpa^F/F^* and *Cebpa*^ΔSftpc^ cells. Each data set was SCTransform-normalized and the top 3,000 highly variable genes (HVGs) across cells were selected.

Principal component analysis (PCA) and UMAP were performed on the integrated object to identify cell clusters. UMAP was performed using the first 30 PCA dimensions. Cell populations in whole lung tissue were annotated using lung cells from the Mouse Cell Atlas 3.0 as a reference (63) using Seurat. Epithelial cells were subset and reanalyzed with a clustering resolution of 0.1, and cell-type annotations were assigned manually to each cluster based on expression of canonical marker genes.

Differential expression testing was performed using MAST (64). Pseuodotime trajectories were constructed using the Monocle 3 R package (65). Transcriptional noise was measured by calculating the Fano factor for each gene using SCTransform-normalized count values across all epithelial cells. Pathway analysis was performed using DAVID Gene Ontology analysis (https://david.ncifcrf.gov/home.jsp).

For publicly available scRNA-Seq analysis, downregulate gene lists are extracted from original papers and are included in Supplemental Table 1. Venn diagrams were prepared using the webtool Venny (https://bioinfogp.cnb.csic.es/tools/venny/index.html). Violin plots of CEBPA expression were constructed using the Monocle 3 R package. The upstream regulator analysis was performed using Ingenuity Upstream Regulator Analysis in IPA (QIAGEN) and are available in Supplemental Table 2. UMAP was generated directly from (http://www.ipfcellatlas.com/).

### Statistics.

Data are presented as means ± SEM. Differences between conditions were examined with GraphPad Prism 9 using an unpaired, 2-tailed Student’s *t* test with Welch’s correction or a nonparametric Mann–Whitney *U* test for experiments with *n* < 10. *P* <0.05 was considered significant.

### Study approval.

All experiments with human tissue samples were performed under protocols approved by IRB at Mayo Clinic. All mouse experiments were conducted under protocols approved by the IACUCs of Mayo Clinic and the University of Minnesota.

### Data availability.

RNA-Seq raw data have been deposited to Gene Expression Omnibus (GEO) database with the accession no. GSE264629. Values for graphs in the figures and supplemental figures are provided in the Supporting Data Values file.

## Author contributions

QT contributed funding acquisition, conceptualization, data acquisition and analysis, methodology, and manuscript writing and editing. JHW contributed data acquisition, methodology and data analysis, and manuscript editing. SS contributed methodology, data acquisition, and analysis. SRD contributed data acquisition and analysis as well as manuscript editing. JAM contributed methodology, data acquisition, and analysis. KMC contributed methodology, data acquisition, and analysis. YL contributed methodology and data acquisition. YSP contributed funding acquisition and intellectual input. DJT contributed funding acquisition, conceptualization, intellectual input, and manuscript writing and editing.

## Supplementary Material

Supplemental data

Unedited blot and gel images

Supporting data values

## Figures and Tables

**Table 1:**
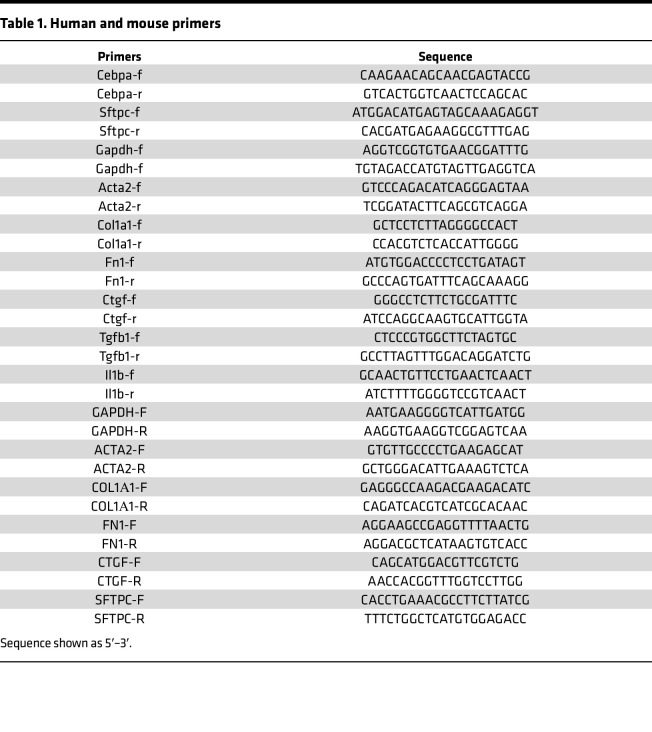
Human and mouse primers

## Version 1 07/16/2024

In-Press Preview

## Version 2 08/22/2024

Electronic publication
