# Supplementary material for: Targeting CEBPA to restore cellular identity and tissue homeostasis in pulmonary fibrosis
Source: JCI Insight. 2024 Jul 16;9(16):e175290. doi: 10.1172/jci.insight.175290 (PMC11343593; doi:10.1172/jci.insight.175290)
Supplement: Supplemental data [file jciinsight-9-175290-s231.pdf]

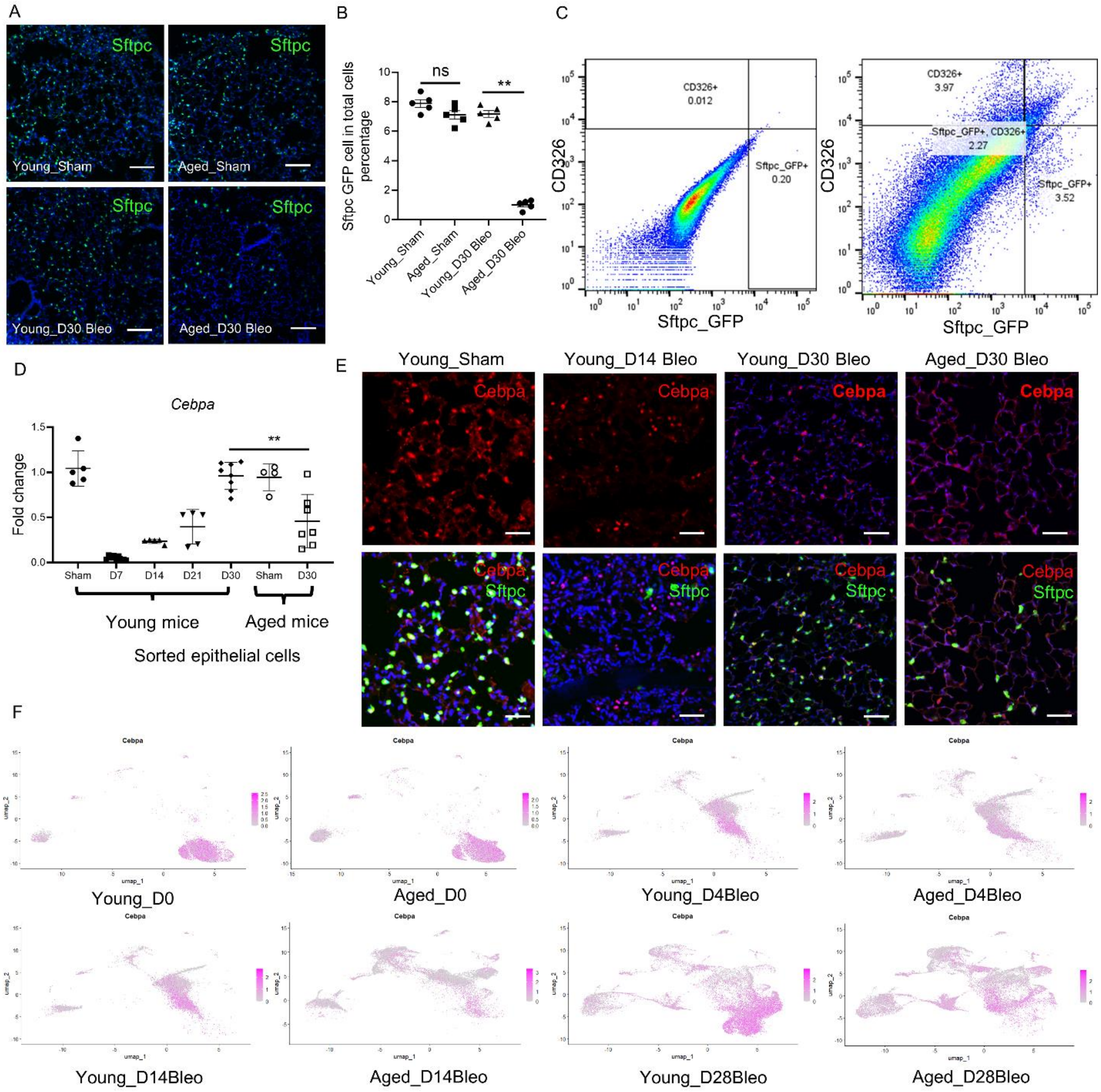

Figure S1. (A) Representative images of AT2 cells represented by Sftpc expression in young (2-month-old; n=5 per timepoint) and aged (18-month-old; n=5 per timepoint) mice 30 days post-bleomycin injury, as lung fibrosis was resolving. DAPI= blue, Sftpc=green. Scale bar=200 μM. (B) AT2 cells were quantified as percentage of total cells in the lung from 30 days post-bleomycin injury between young and aged mice. (C) Representative flow cytometry analysis of CD326+ cells and Sftpc_GFP cells in the mice lung. (D) Quantitative PCR of *Cebpa* gene expression performed on flow cytometry–sorted epithelial cells (CD326+, CD45-, CD31-) isolated from the lungs of young and aged mice. (E) Representative immunostaining images of

Cebpa expression in young (2-month-old; n=5 per timepoint) and aged (18-month-old; n=5 per timepoint) mice after bleomycin injury, as lung fibrosis was resolving. (F) UMAP plot of *Cebpa* expression at different time points (D0, D4, D14, D28) after bleomycin injury in the lung of young and aged mice from reanalysis of GSE157995. Data were analyzed using a Mann–Whitney U test. Statistical significance: *P<0.05, **P<0.01, ns = not significant.

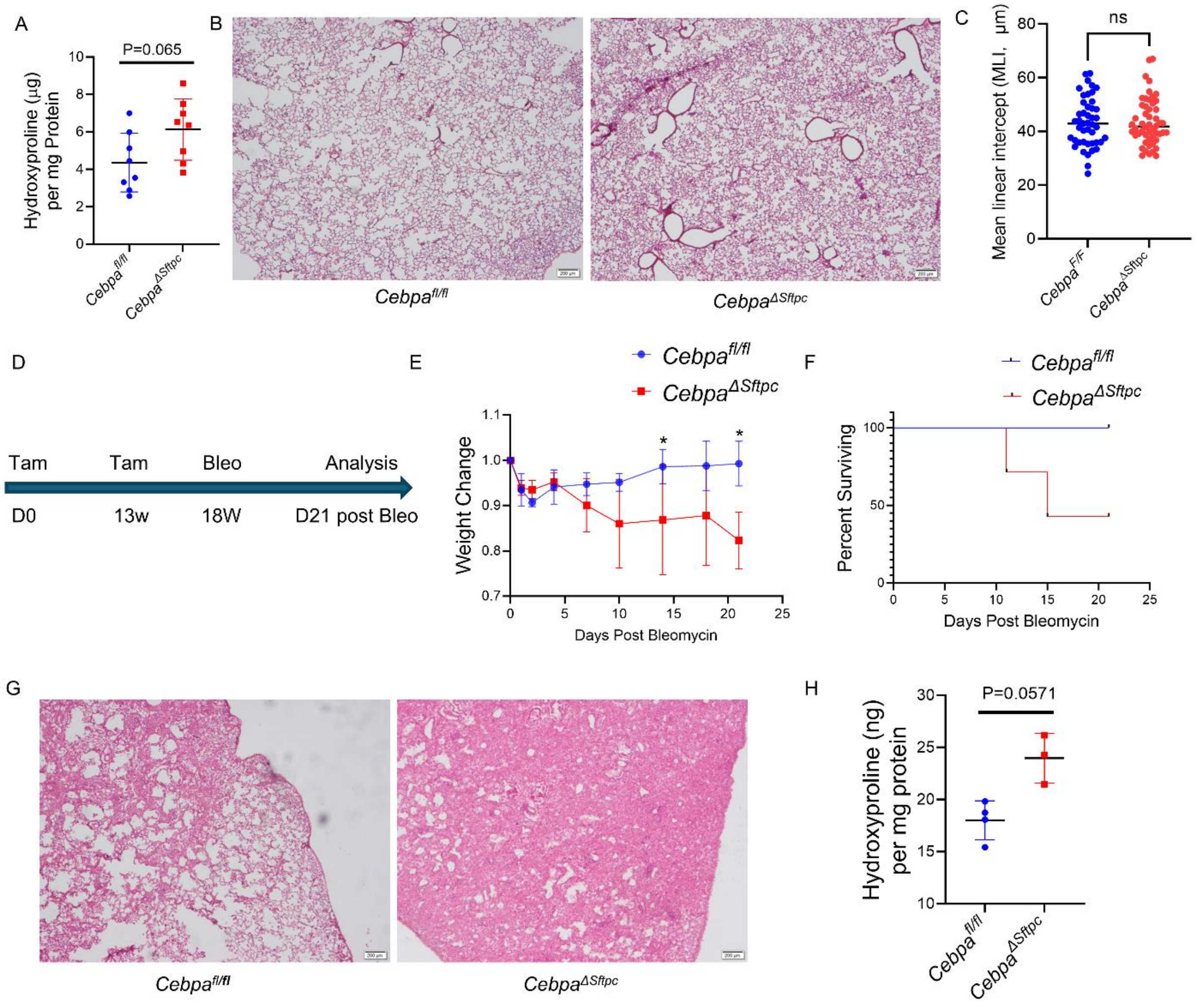

Figure S2. (A) Hydroxyproline assay showing collagen deposition in the lungs of *Cebpa*$^{\Delta Sftpc}$ (n=8) and *Cebpa*$^{fl/fl}$ (n=8) mice 4 weeks after tamoxifen treatment to delete the *Cebpa* gene in *Cebpa*$^{\Delta Sftpc}$ mice. (B) Representative hematoxylin and eosin (H&E) staining images showing lung sections from *Cebpa*$^{\Delta Sftpc}$ (n=4) and *Cebpa*$^{fl/fl}$ (n=4) mice 4 weeks after tamoxifen treatment. Scale bar=200 µM. (C) Mean linear intercept measurement of H&E staining images from *Cebpa*$^{\Delta Sftpc}$ (n=4) and *Cebpa*$^{fl/fl}$ (n=4) mice 4 weeks after tamoxifen treatment. (D) Schematic showing timeline for tamoxifen treatment (to delete *Cebpa* in *Cebpa*$^{\Delta Sftpc}$ mice), bleomycin treatment (to induce fibrosis), and analysis for *Cebpa*$^{\Delta Sftpc}$ (n=7) and *Cebpa*$^{fl/fl}$ mice (n=5). (E) Weight and (F) survival of *Cebpa*$^{\Delta Sftpc}$ and *Cebpa*$^{fl/fl}$ mice from (A) post-bleomycin treatment. (G) Representative hematoxylin and eosin (H&E) staining images showing lung sections from *Cebpa*$^{\Delta Sftpc}$ (n=3) and *Cebpa*$^{fl/fl}$ mice (n=3) 21 days post-bleomycin treatment. (H)

Hydroxyproline assay for *Cebpa*$^{\Delta Sftpc}$ (n=3) and *Cebpa*$^{fl/fl}$ mice (n=4), 21 days post-bleomycin treatment. Data were analyzed using a Mann–Whitney U test. Statistical significance: *P<0.05, ns = not significant.

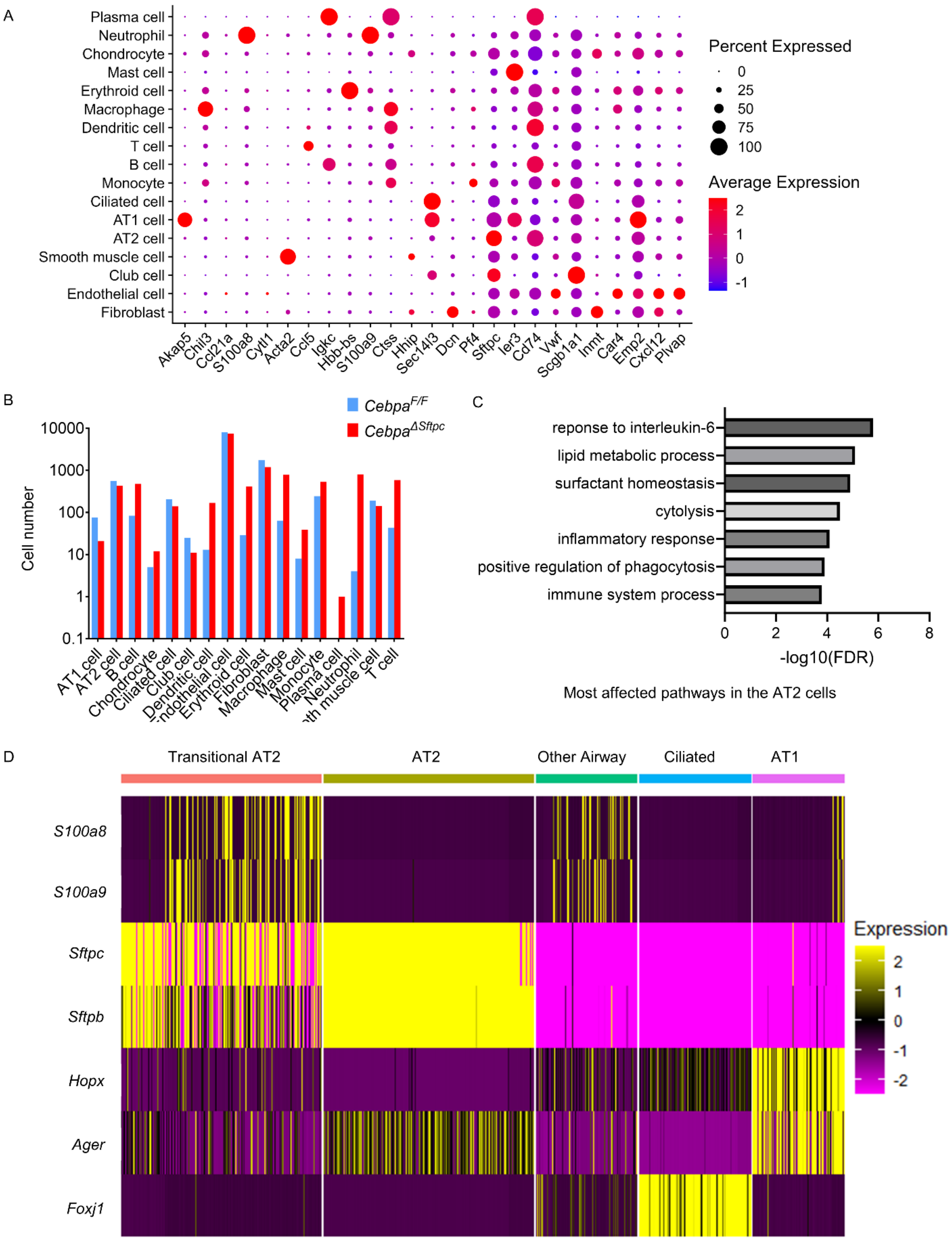
A
Plasma cell
Neutrophil
Chondrocyte
Mast cell
Erythroid cell
Macrophage
Dendritic cell
T cell
B cell
Monocyte
Ciliated cell
AT1 cell
AT2 cell
Smooth muscle cell
Club cell
Endothelial cell
Fibroblast
Akap5 Chil3 Ccl21a S100a8 Cytl1 Acta2 Ccl5 Igkc Hbb-bs S100a9 Ctss Hhip Sec14l3 Dcn Pf4 Sftpc Ier3 Cd74 Vwf Scgb1a1 Inmt Car4 Emp2 Cxcl12 Plvap
Percent Expressed
0
25
50
75
100
Average Expression
2
1
0
-1
B
Cebpa^F/F
Cebpa^ΔSftpc
Cell number
10000
1000
100
10
1
0.1
AT1 cell AT2 cell B cell Chondrocyte Ciliated cell Club cell Dendritic cell Endothelial cell Erythroid cell Fibroblast Macrophage Mast cell Monocyte Plasma cell Neutrophil Smooth muscle cell T cell
C
reponse to interleukin-6
lipid metabolic process
surfactant homeostasis
cytolysis
inflammatory response
positive regulation of phagocytosis
immune system process
0 2 4 6 8
-log10(FDR)
Most affected pathways in the AT2 cells
D
Transitional AT2
AT2
Other Airway
Ciliated
AT1
S100a8
S100a9
Sftpc
Sftpb
Hopx
Ager
Foxj1
Expression
2
1
0
-1
-2

Figure S3. Dot plots showing the marker genes for each cluster and their cellular annotations for both *Cebpa*$^{\Delta Sftpc}$ and *Cebpa*$^{fl/fl}$ mice in the scRNA-seq analysis. (B) Cell numbers for each cluster from the *Cebpa*$^{\Delta Sftpc}$ and *Cebpa*$^{fl/fl}$ mouse samples shown in Fig 3A. (C) Pathway analysis of list of genes significantly downregulated in AT2 cells from *Cebpa*$^{\Delta Sftpc}$ vs *Cebpa*$^{fl/fl}$ mice. (D) Heatmap showing the marker genes for each cluster from all the epithelial cells for for both *Cebpa*$^{\Delta Sftpc}$ and *Cebpa*$^{fl/fl}$ mice.

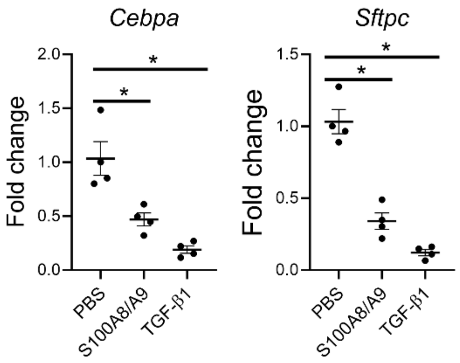

Figure S4. Quantitative PCR for *Cebpa* and *Sftpc* trancripts in *Sftpc*-gfp mouse lung organoids (n=4) treated with the recombinant proteins S100A8/A9 (100 ng/ml) and TGF-β1 (10 ng/ml). Cells were analyzed 72hours after treatment. Data were analyzed using a Mann–Whitney U test. *P<0.05.

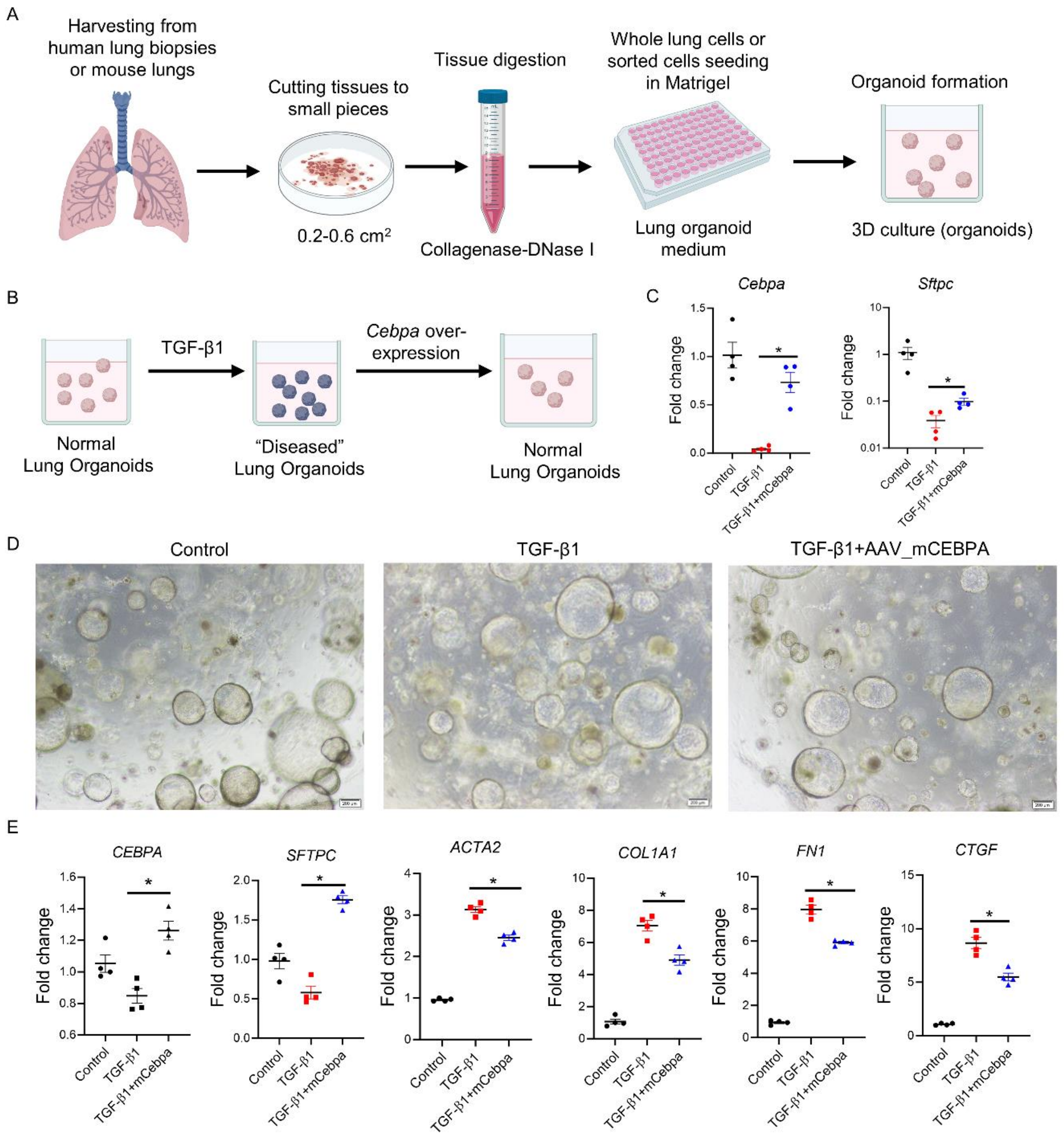

Figure S5. (A) Schematic showing how lung organoids are generated. Created with BioRender.com. (B) Schematic showing how lung organoids are used for lung fibrosis model and test Cebpa overexpression on them. Created with BioRender.com. (C) Quantitative PCR for *Cebpa* and *Sftpc* from mouse epithelial cells derived lung organoids treated with TGF-β1, with or without *Cebpa* overexpression. (D) Representative bright field image of organoids formed from human lung tissue (n=4). (E) Quantitative PCR for profibrotic gene and *Sftpc* transcripts from human lung organoids treated with TGF-β1, with or without AAV9-mCebpa treatment. Data were analyzed using a Mann–Whitney U test. *$P<0.05$.

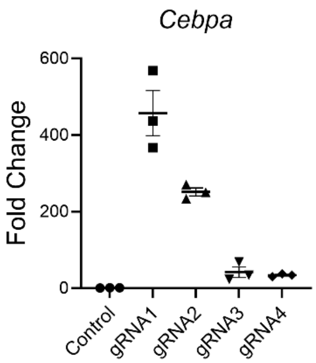

Figure S6. Quantitative PCR for *Cebpa* transcripts in Cas9+ CD326+ cells harvested from Cas9 mice and transfected with a dgRNA-MPH plasmid containing each of four individual gRNAs targeting the *Cebpa* promoter, as shown in Figure 6G. Cells were analyzed 48 hours after transfection.

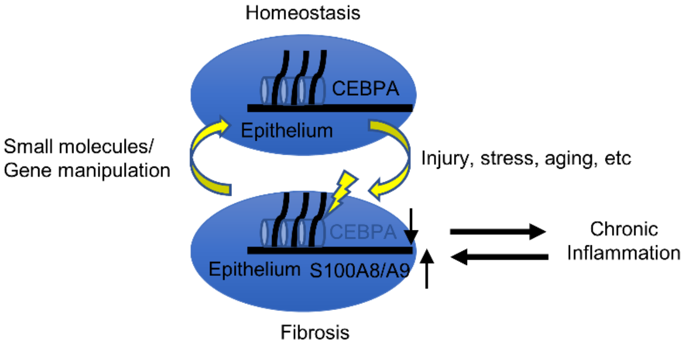

Figure S7. Schematic showing how CEBPA in AT2 cells (blue ovals) mediates cellular identity and tissue homeostasis.
