# Supplementary figures and images for: Targeting CEBPA to restore cellular identity and tissue homeostasis in pulmonary fibrosis
Source: JCI Insight. 2024 Jul 16;9(16):e175290. doi: 10.1172/jci.insight.175290 (PMC11343593; doi:10.1172/jci.insight.175290)

Figure 2D

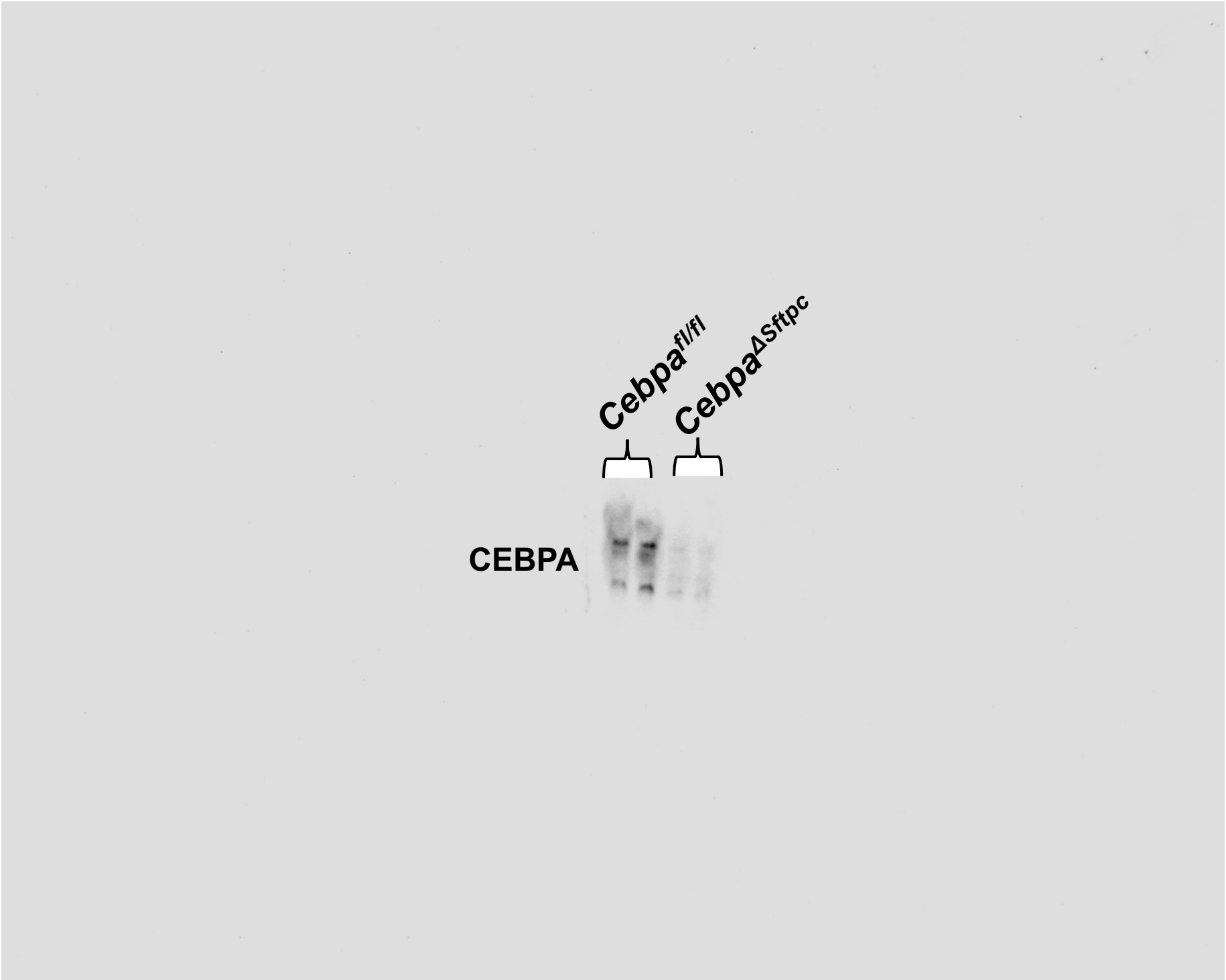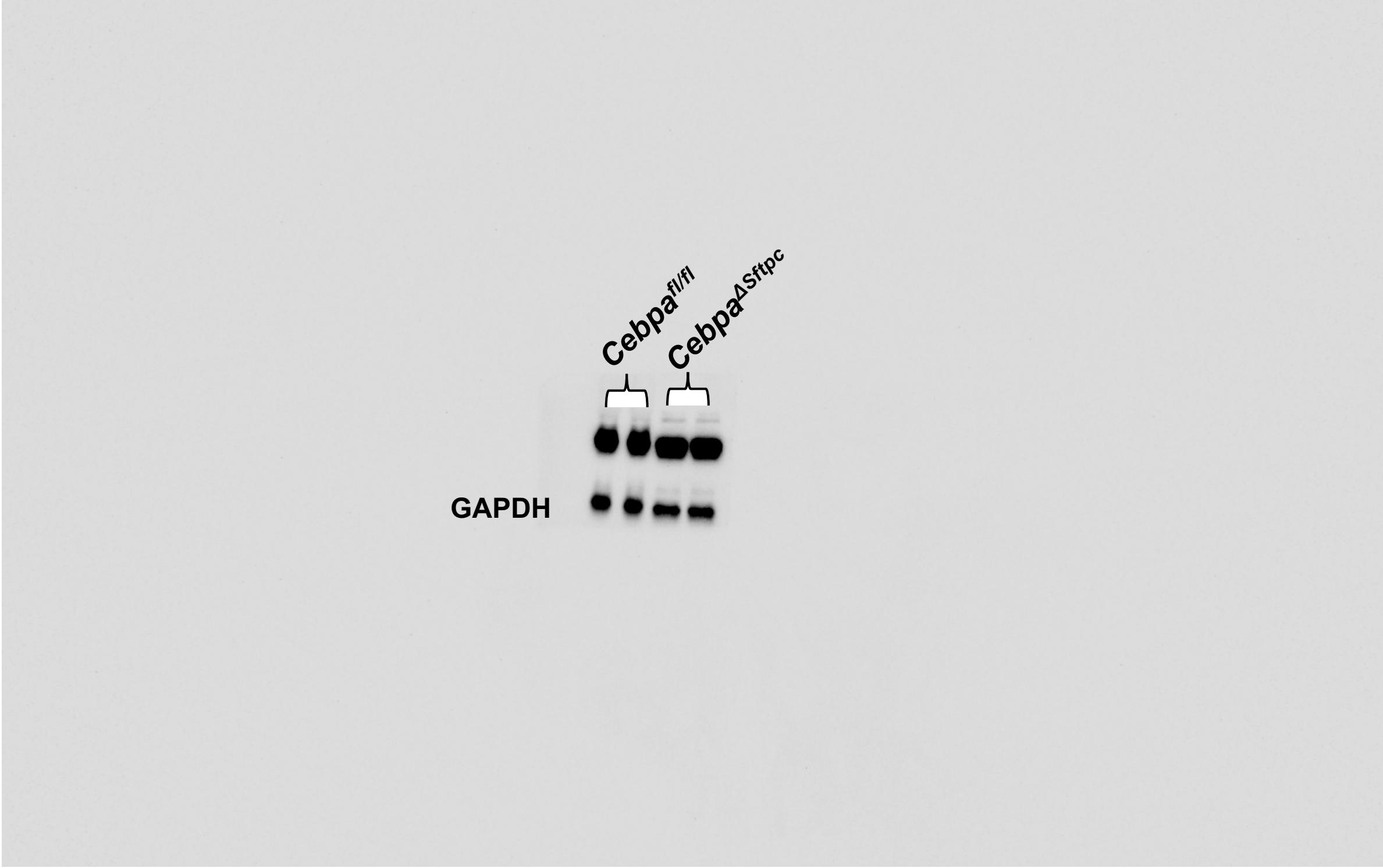

Figure 2H

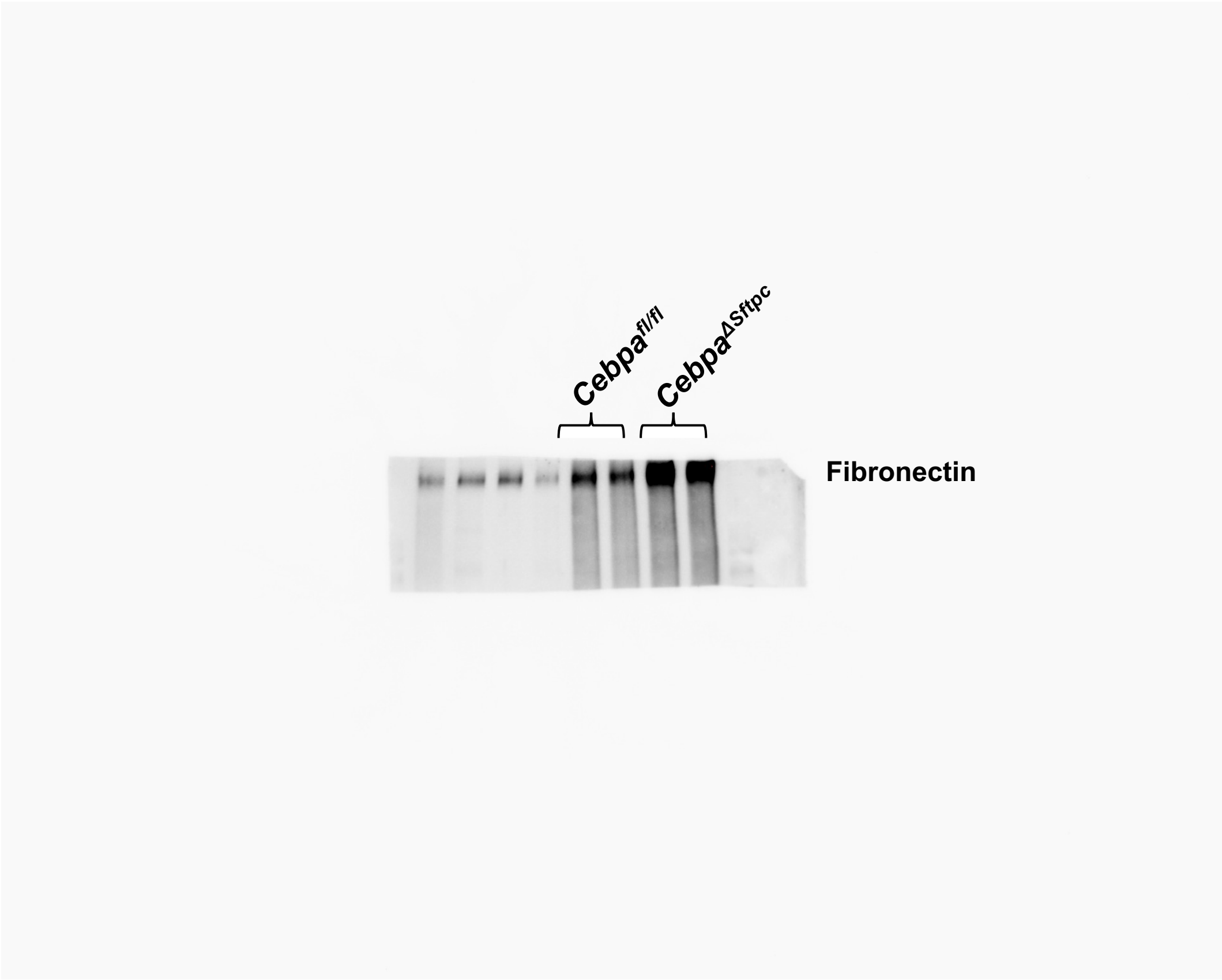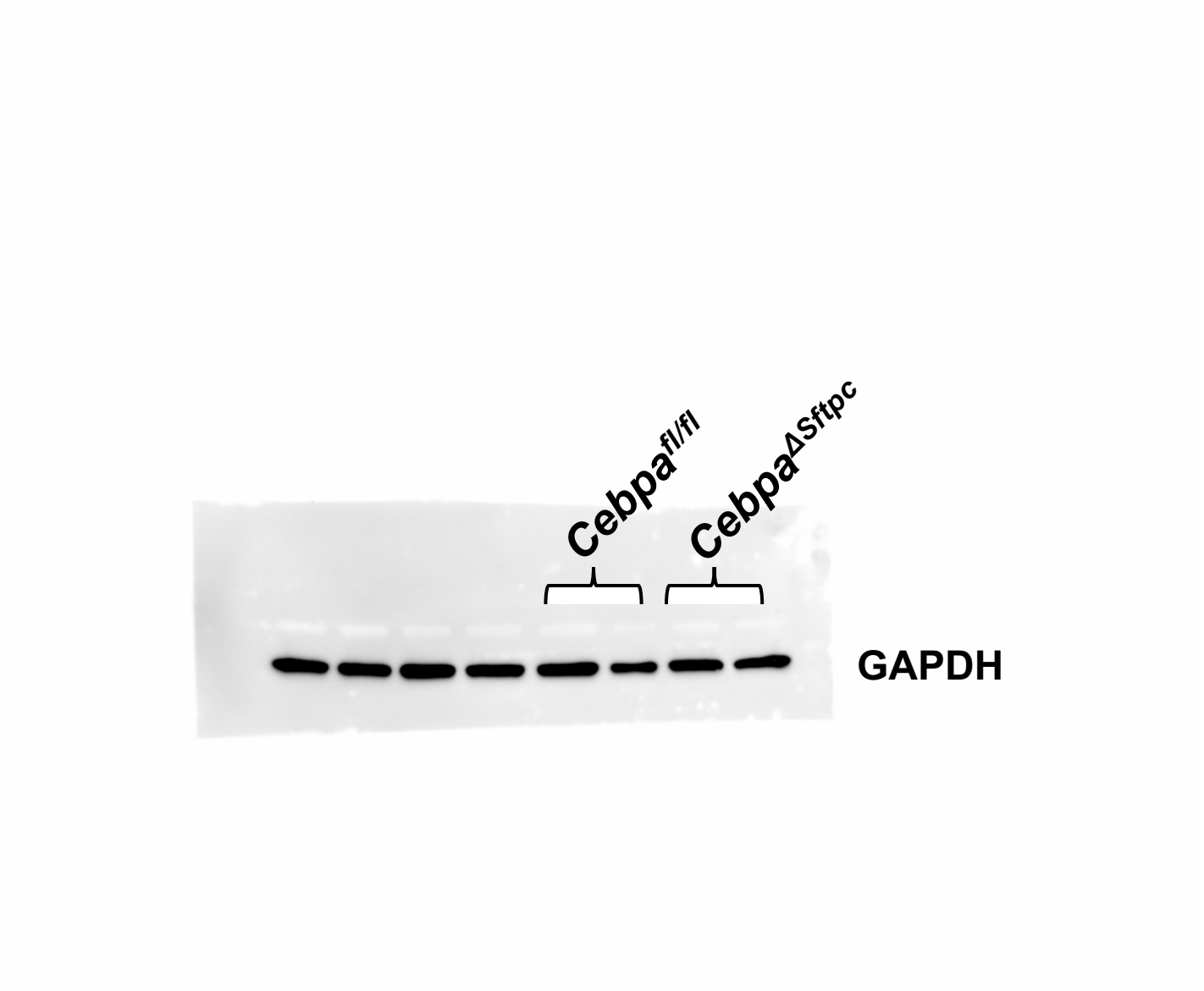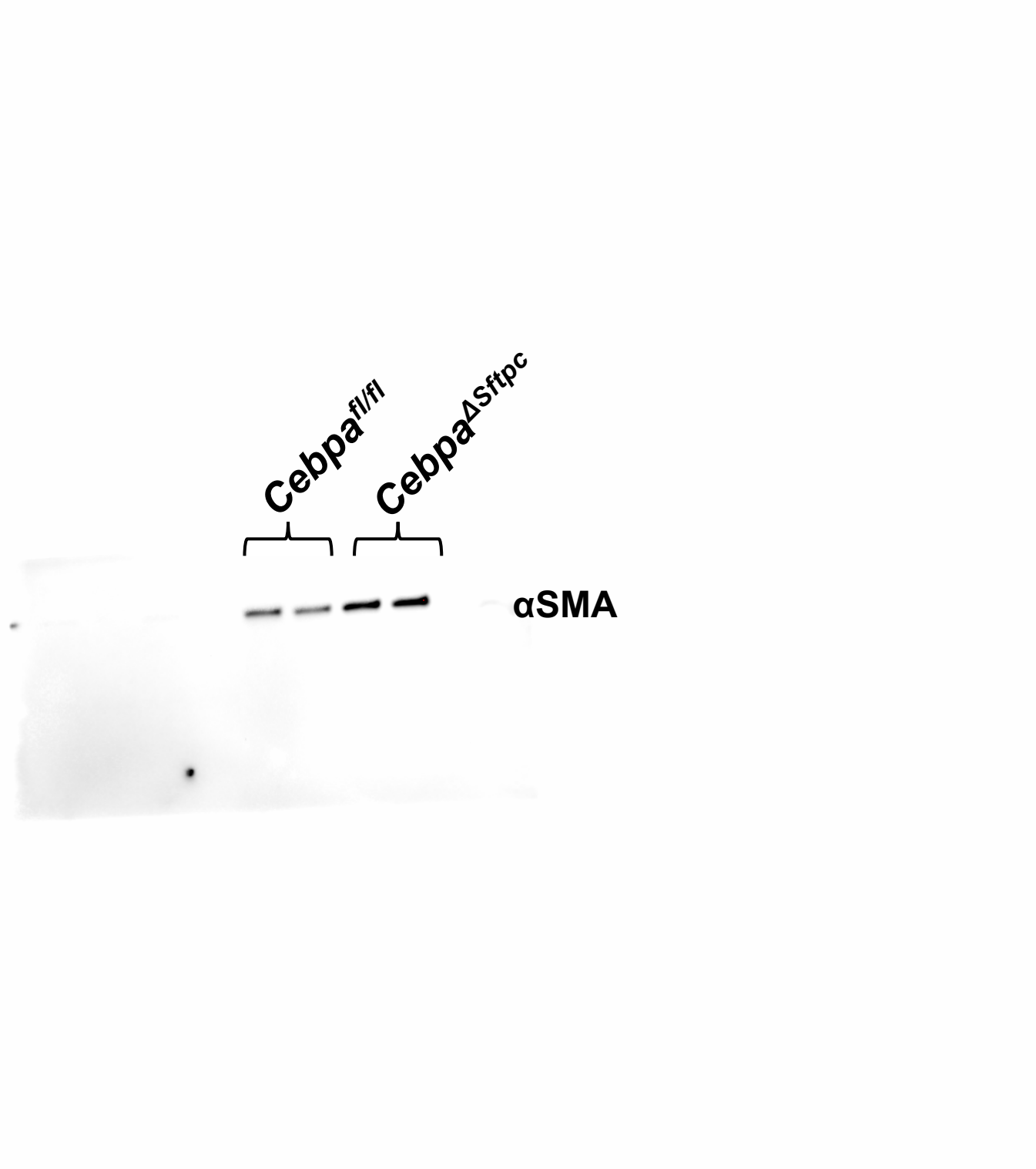

Figure 6I

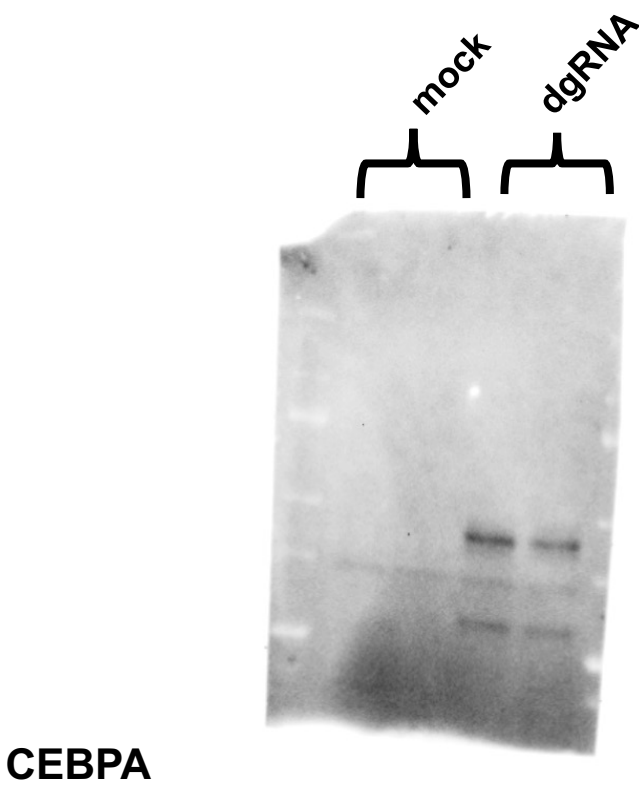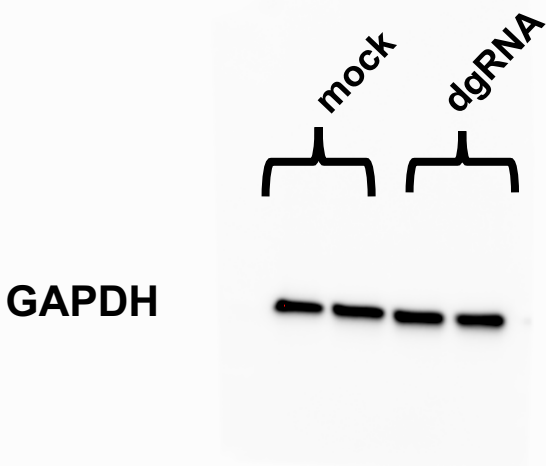

Supplement: Unedited blot and gel images [file jciinsight-9-175290-s232.pdf]
